# The endocytosis of oxidized LDL via the activation of the angiotensin II type 1 receptor

**DOI:** 10.1016/j.isci.2021.102076

**Published:** 2021-01-21

**Authors:** Toshimasa Takahashi, Yibin Huang, Koichi Yamamoto, Go Hamano, Akemi Kakino, Fei Kang, Yuki Imaizumi, Hikari Takeshita, Yoichi Nozato, Satoko Nozato, Serina Yokoyama, Motonori Nagasawa, Tatsuo Kawai, Masao Takeda, Taku Fujimoto, Kazuhiro Hongyo, Futoshi Nakagami, Hiroshi Akasaka, Yoichi Takami, Yasushi Takeya, Ken Sugimoto, Herbert Y. Gaisano, Tatsuya Sawamura, Hiromi Rakugi

**Affiliations:** 1 Department of Geriatric and General Medicine, Osaka University Graduate School of Medicine, 2-2 Yamadaoka, Suita, Osaka, 565-0871, Japan; 2 Department of Medicine, University of Toronto, Toronto, Ontario M5S1A8, Canada; 3 Department of Molecular Pathophysiology, Shinshu University Graduate School of Medicine, Matsumoto, Nagano 390-8621, Japan

**Keywords:** Molecular Biology, Cell Biology

## Abstract

Arrestin-dependent activation of a G-protein-coupled receptor (GPCR) triggers endocytotic internalization of the receptor complex. We analyzed the interaction between the pattern recognition receptor (PRR) lectin-like oxidized low-density lipoprotein (oxLDL) receptor (LOX-1) and the GPCR angiotensin II type 1 receptor (AT1) to report a hitherto unidentified mechanism whereby internalization of the GPCR mediates cellular endocytosis of the PRR ligand. Using genetically modified Chinese hamster ovary cells, we found that oxLDL activates Gαi but not the Gαq pathway of AT1 in the presence of LOX-1. Endocytosis of the oxLDL-LOX-1 complex through the AT1-β-arrestin pathway was demonstrated by real-time imaging of the membrane dynamics of LOX-1 and visualization of endocytosis of oxLDL. Finally, this endocytotic pathway involving GPCR kinases (GRKs), β-arrestin, and clathrin is relevant in accumulating oxLDL in human vascular endothelial cells. Together, our findings indicate that oxLDL activates selective G proteins and β-arrestin-dependent internalization of AT1, whereby the oxLDL-LOX-1 complex undergoes endocytosis.

## Highlights

• The binding of oxidized LDL (oxLDL) to LOX-1 induces selective activation of AT1
• oxLDL and angiotensin II additively or competitively activate AT1 in different cells
• oxLDL promotes β-arrestin-dependent internalization of oxLDL-LOX-1-AT1 complex

## Graphical abstract

**Figure:**
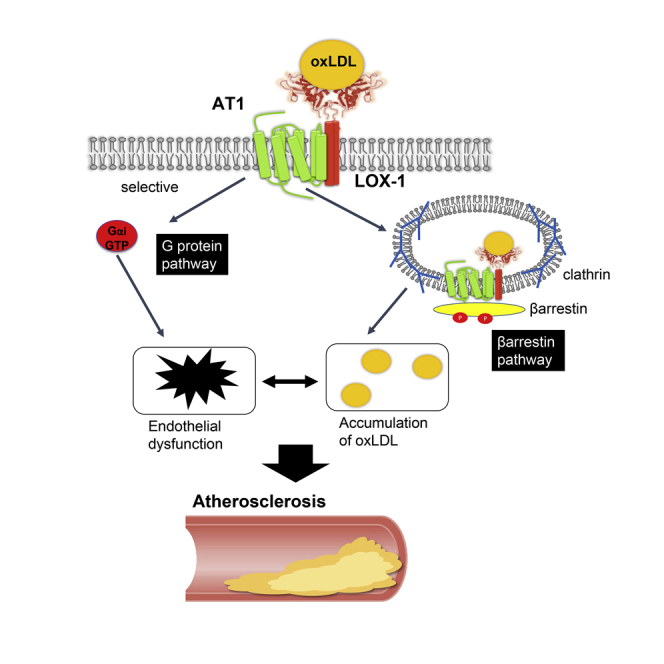

## Introduction

Seven-transmembrane G-protein-coupled receptors (GPCRs) have long been attractive targets for drug discovery and development, as evidenced by the fact that about one-third of all drug targets are GPCRs (Santos et al., 2017). In addition to canonical activation by binding of the cognate ligand, recent understanding of the allosteric activation pathways of GPCRs has revealed the diversity in the physiological and pathological roles of this receptor family (Gentry et al., 2015; Khoury et al., 2014; Wootten et al., 2013). The allosteric activation of GPCRs is primarily induced by ligand binding to the allosteric ligand-binding domains of the GPCR (Gentry et al., 2015; Khoury et al., 2014; Wootten et al., 2013). The allosteric ligands of GPCRs trigger a cellular response distinct from that elicited by orthosteric ligands by differentially regulating activation of G proteins and arrestins, the latter primarily mediating cellular trafficking of receptor complexes (Jean-Charles et al., 2017; Ranjan et al., 2017; Smith and Rajagopal, 2016).

We recently reported that the angiotensin II type 1 receptor (AT1), a member of the class A GPCR family, forms a complex with the single transmembrane lectin-like oxidized low-density lipoprotein (oxLDL) receptor LOX-1 on the cellular membrane and thereby mediates oxLDL-induced AT1 activation, leading to vascular endothelial dysfunction in mice (Yamamoto et al., 2015). It has been increasingly recognized that heterodimerization of GPCRs could bias the signaling pathway of the partner receptor (Ferre et al., 2014; Forrester et al., 2018). It has also been reported that ligand binding to GPCRs triggers signal transduction modified by interactions with non-GPCRs, such as ion channels (Hermosilla et al., 2017; Shenoy and Lefkowitz, 2011; Shukla et al., 2010). However, our previous study is the only example of ligand binding to single transmembrane receptor-induced activation of an adjacent GPCR in the oligomeric complex (Yamamoto et al., 2015). Therefore, the precise molecular pathways that are activated upon stimulation remain largely undetermined in this case.

The binding of AT1 to its original ligand angiotensin II (Ang II) triggers various cellular responses, primarily by activating specific G proteins, including Gαq/11, Gαi/0, and Gα12/13, with Gαq/11 being responsible for Ang II-induced arterial contraction (Berk, 1999; Hunyady and Catt, 2006). Since oxLDL is not a potent vasoconstrictor, it is conceivable that the G protein activation of AT1 by oxLDL is distinct from that by Ang II.

Internalization and intracellular trafficking of AT1 mediated by its interaction with β-arrestins (ARRBs) are the cellular responses of desensitization to prolonged Ang II stimulation (Hunyady, 1999; Hunyady et al., 2000). However, this also functions as a transport system for molecules adjacent to the receptor. Accumulation of oxLDL in atherosclerotic lesions is a major observation in the development of atherosclerosis (Steinberg, 1997; Steinberg and Witztum, 2010). Considering the pathophysiological importance of the intracellular accumulation of oxLDL in atherosclerosis, it is worth clarifying whether oxLDL binding to LOX-1 can trigger AT1 internalization, translocation of the oxLDL-LOX-1-AT1 complex, and eventually lead to intracellular accumulation of oxLDL.

In the present study, we investigated two topics: (1) whether oxLDL induces the activation of the same G protein as Ang II and (2) whether oxLDL-induced AT1 activation causes the cellular accumulation of oxLDL.

## Results

### oxLDL-induced activation of AT1 is G protein selective

**Figure 1:**
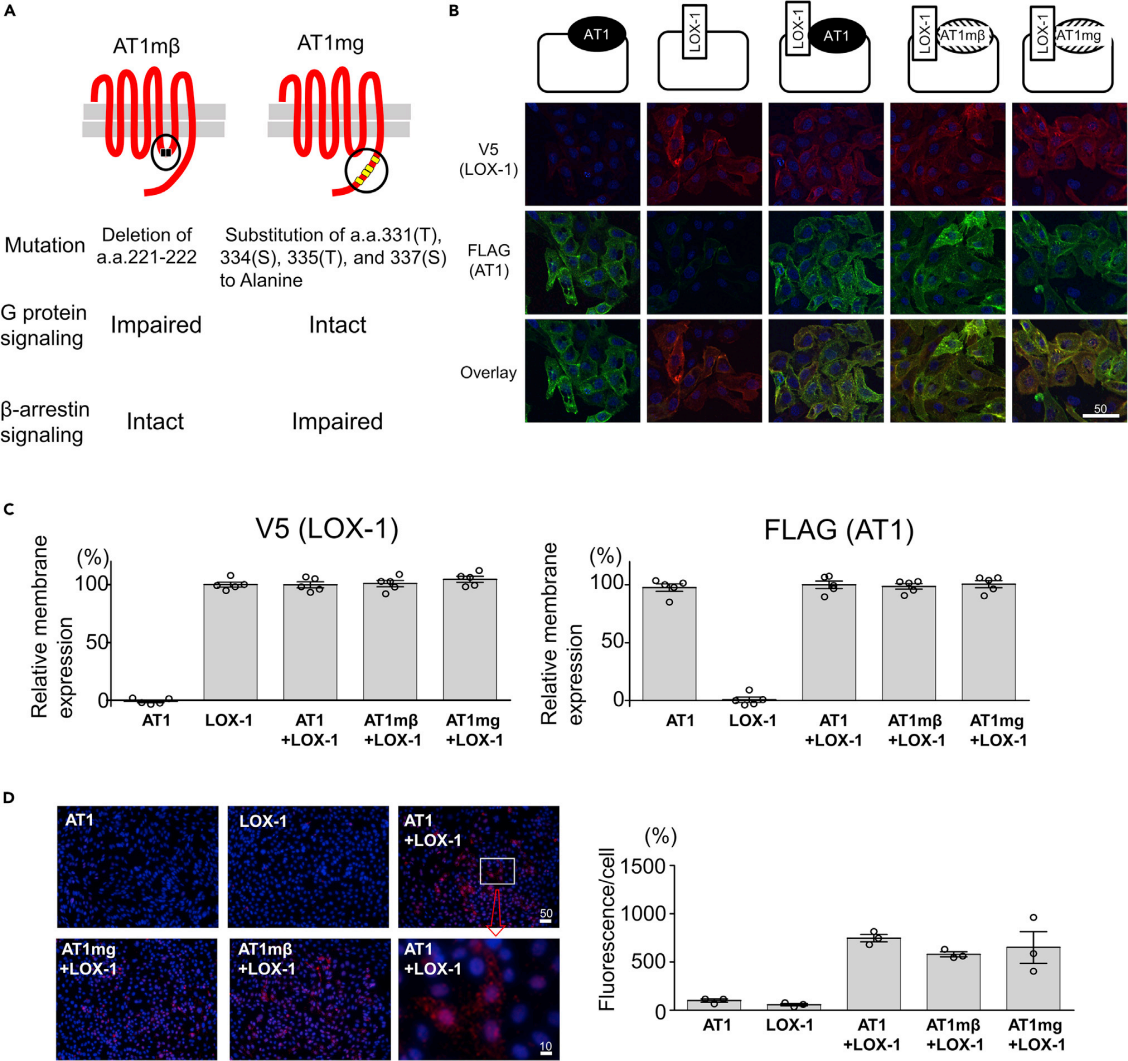
Generation of genetically engineered CHO cells expressing LOX-1 and/or AT1 or biased AT1 mutants (A) Overview of two biased AT1 mutants. AT1mβ, AT1 mutant with β-arrestin-biased signaling; AT1mg, AT1 mutant with G-protein-biased signaling. (B) Immunofluorescence of CHO cells stably expressing V5-tagged LOX-1 and/or FLAG-tagged AT1 or biased AT1 mutants using anti-V5 or anti-FLAG antibodies. Scale bar (μm). (C) Quantification of membrane-expressing V5-tagged LOX-1 and FLAG-tagged AT1 or biased AT1 mutants by cell-based ELISA. Absorbance in CHO cells expressing V5-tagged LOX-1 and FLAG-tagged AT1 (CHO-LOX-1-AT1) was normalized to 100% (n = 5, each). Data are represented as mean ± SEM. The differences were determined by one-way analysis of variance (ANOVA) with Bonferroni correction. ^∗^p < 0.01 vs. the other types of cells. (D) *In situ* proximity ligation assay (PLA) of CHO cells stably expressing V5-tagged LOX-1 and/or FLAG-tagged AT1 or biased AT1 mutants using anti-V5 and anti-FLAG antibodies. Scale bar (μm). The graph indicates the fluorescence/number of nuclei (% of that in CHO-AT1) (n = 3, each) Data are represented as mean ± SEM. The differences were determined by one-way ANOVA with Bonferroni correction. ^∗^p < 0.01 vs.CHO-AT1, CHO-LOX-1, †p < 0.05 vs.CHO-AT1, p < 0.01 vs. CHO-LOX-1.

In this study, we used five different genetically engineered Chinese hamster ovary (CHO) cells that endogenously express neither LOX-1 nor AT1 to individually analyze the G protein and β-arrestin pathways of oxLDL-induced AT1 activation. CHO cells expressing either human (h)AT1R or hLOX1 (CHO-AT1 and CHO-LOX-1, respectively), CHO cells expressing both hLOX-1 and hAT1 (CHO-LOX-1-AT1), CHO cells expressing both hLOX-1 and mutated hAT1 with impaired ability to activate G protein (Haendeler et al., 2000) (CHO-LOX-1-AT1 mutant with β-arrestin-biased signaling [CHO-LOX-1-AT1mβ]), and CHO cells expressing both hLOX-1 and mutated hAT1 with impaired ability to activate β-arrestin (CHO-LOX-1-AT1 mutant with G-protein-biased signaling [CHO-LOX-1-AT1mg]) (Qian et al., 2001) (Figures 1A and 1B) were used. There was no difference in the magnitude of membrane expression of these receptors among the respective cells, as assessed by cell-based enzyme-linked immunosorbent assay (ELISA) in non-permeabilized conditions (Figure 1C).

We previously found that the proximity between LOX-1 and AT1 on the cellular membrane was absent between LOX-1 and the Ang II type receptor (AT2), which is the isoform of AT1, in an *in situ* proximity ligation assay (PLA), indicating that LOX-1 specifically binds to AT1 (Yamamoto et al., 2015). Similar red fluorescence intensity was observed in *in situ* PLA in non-permeabilized conditions in CHO-LOX-1-AT1, CHO-LOX-1-AT1mβ, and CHO-LOX-1-AT1mg, suggesting that these AT1 mutants interact with LOX-1, similar to the interaction with intact AT1 on the cellular surface (Figure 1D).

**Figure 2:**
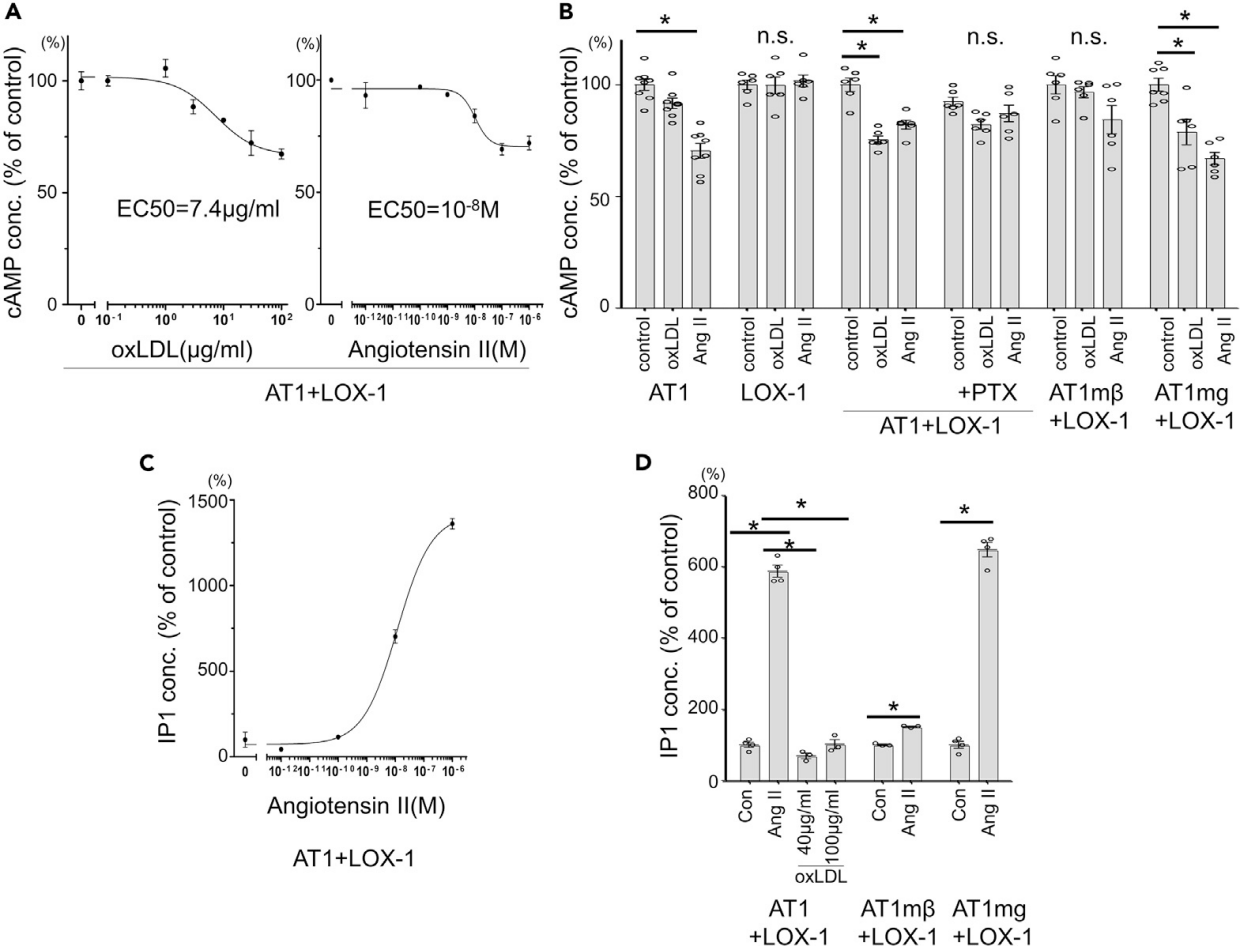
Assessment of Gαi and Gq signaling in genetically engineered CHO cells by quantifying reduction in Forskolin-induced cAMP accumulation and increase in IP1 production, respectively (A) Dose-dependent response in cyclic adenosine monophosphate (cAMP) concentration in response to oxLDL or angiotensin II in CHO-LOX-1-AT1. Cells were treated with 1 μM forskolin to induce cAMP accumulation (n = 4, each). Data are represented as mean ± SEM. (B) cAMP concentration in response to vehicle, 40 μg/mL oxLDL or 10^−8^ M angiotensin II in genetically engineered CHO cells. Gi inhibitor pertussis toxin (PTX) was pretreated at 25 ng/mL for 12 h before stimulation. The concentration in control-treated wells in each cell type was normalized to 100% (n = 6–8). Data are represented as mean ± SEM. The differences were determined by one-way analysis of variance (ANOVA) with Bonferroni correction. ∗p < 0.01. (C) Dose-dependent response in inositol monophosphate (IP1) concentration in response to angiotensin II in CHO-LOX-1-AT1, expressed as percentage of IP1 concentration in the absence of angiotensin II (n = 4, each). Data are represented as mean ± SEM. (D) IP1 concentration in response to vehicle, angiotensin II (10^−7^M), or oxLDL in genetically engineered CHO cells. The concentration in control wells of each cell type was normalized to 100% (n = 4, each). Data are represented as mean ± SEM. The differences were determined by one-way ANOVA with Bonferroni correction. ∗p < 0.01.

These cells were used to investigate whether oxLDL could activate Gαi and Gαq, which interact with AT1, by quantifying the reduction in Forskolin-induced cyclic adenosine monophosphate (cAMP) accumulation and increase in inositol monophosphate (IP1) production, respectively. As shown in Figure 2A, oxLDL and Ang II decreased cAMP in CHO-LOX-1-AT1 in a dose-dependent manner, with an EC_50_ of 7.4 μg/mL and 10^−8^ M, respectively. A reduction in cAMP by 40 μg/mL oxLDL was not observed in either CHO-LOX1 or CHO-LOX-1-AT1 cells treated with the Gαi inhibitor pertussis toxin (PTX) (Figure 2B). G protein dependence of this phenomenon was also confirmed by the reduction in cAMP by oxLDL in CHO-LOX-1-AT1mg but not in CHO-LOX-1-AT1mβ (Figure 2B).

In contrast to the findings regarding Gαi signaling, oxLDL did not induce the activation of Gαq signaling through the LOX-1-AT1-dependent pathway, as shown by the lack of IP1 accumulation in response to high concentrations of oxLDL in CHO-LOX-1-AT1 (Figure 2D). As expected, Ang II dose dependently increased IP1 production in CHO-LOX-1-AT1, and the effect was highly reduced in CHO-LOX-1-AT1mβ but not in CHO-LOX-1-AT1mg (Figures 2C and 2D). These findings suggest that oxLDL-induced activation of AT1 is distinct from the Ang II-induced activation of AT1 in terms of G protein selectivity.

### oxLDL induces cellular inflammation additively with Ang II via the AT1-G-protein-dependent pathway

**Figure 3:**
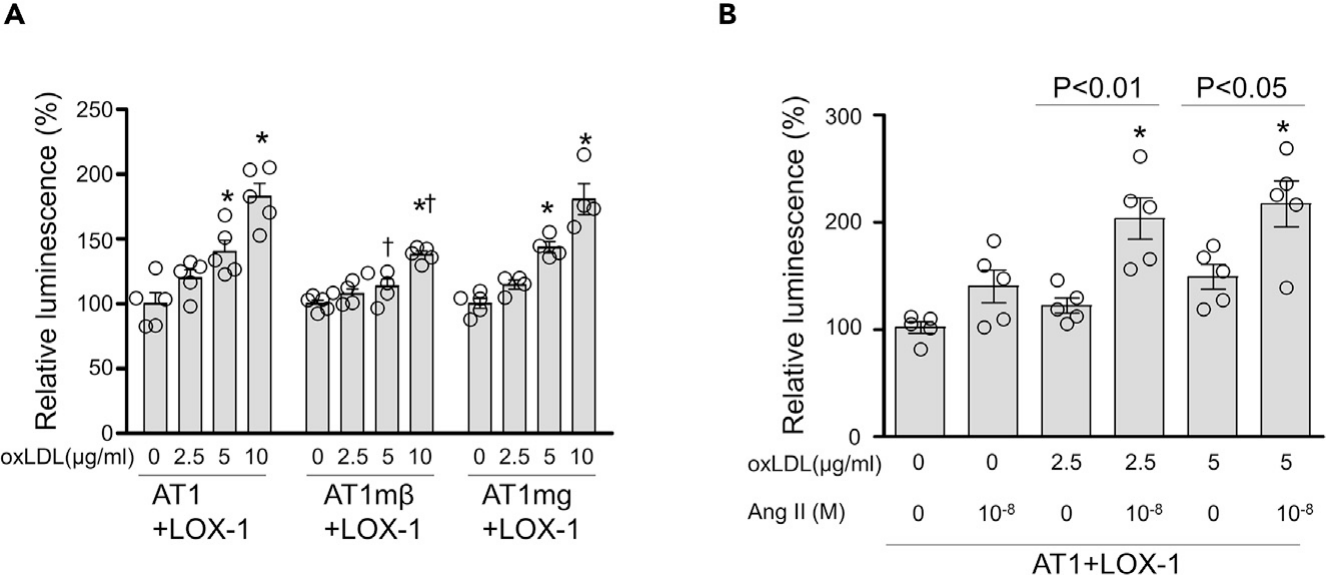
Assessment of NfkB activity in genetically engineered CHO cells (A) NfκB activity detected by a luciferase reporter assay in CHO-LOX-1-AT1, CHO-LOX-1-AT1mβ, and CHO-LOX-1-AT1mg treated with different concentration of oxLDL for 24 hr. The relative luminescence (NfkB-luciferase/control-renilla) in each cell type treated with 0μg/ml oxLDL in each cell type was normalized to 100% (n = 5). Data are represented as mean ± SEM. The differences were determined by one-way analysis of variance (ANOVA) with Bonferroni correction. ^∗^p < 0.05 vs. 0μg/ml oxLDL. †p < 0.05 vs. concentration-matched oxLDL in CHO-LOX-1-AT1mβ. (B) NfκB activity detected by a luciferase reporter assay in CHO-LOX-1-AT1 treated with vehicle, 2.5, 5μg/ml oxLDL, 10^−8^ M Ang II, or oxLDL in combination with Ang II for 24 hr. The relative luminescence (NfkB-luciferase/control-renilla) in each cell type treated with 0μg/ml oxLDL in each cell type was normalized to 100% (n = 5). Data are represented as mean ± SEM. The differences were determined by one-way ANOVA with Bonferroni correction. ^∗^p < 0.01 vs. vehicle treatment.

We assessed cellular inflammation by detecting the activation of NF-κB using the dual luciferase reporter assay system in the genetically engineered CHO cells. As shown in Figure 3A, oxLDL dose dependently increased the activation of NfκB in CHO-LOX-1-AT1 and CHO-LOX-1-AT1mg, and the activation was significantly attenuated in CHO-LOX-1-AT1mβ, suggesting that oxLDL-induced inflammatory response involves G-protein-dependent but not β-arrestin-dependent signaling pathway of AT1. We also found that the activation of NfκB increased prominently in response to the combined treatment of Ang II with oxLDL compared to either treatment alone in CHO-LOX-1-AT1, suggesting that the Ang II-AT1 signaling and the oxLDL-LOX-1-AT1 signaling have additive effect on cellular inflammation in this cell condition (Figure 3B).

### oxLDL induces internalization of LOX-1 and AT1 via a β-arrestin-dependent signaling pathway of AT1

**Figure 4:**
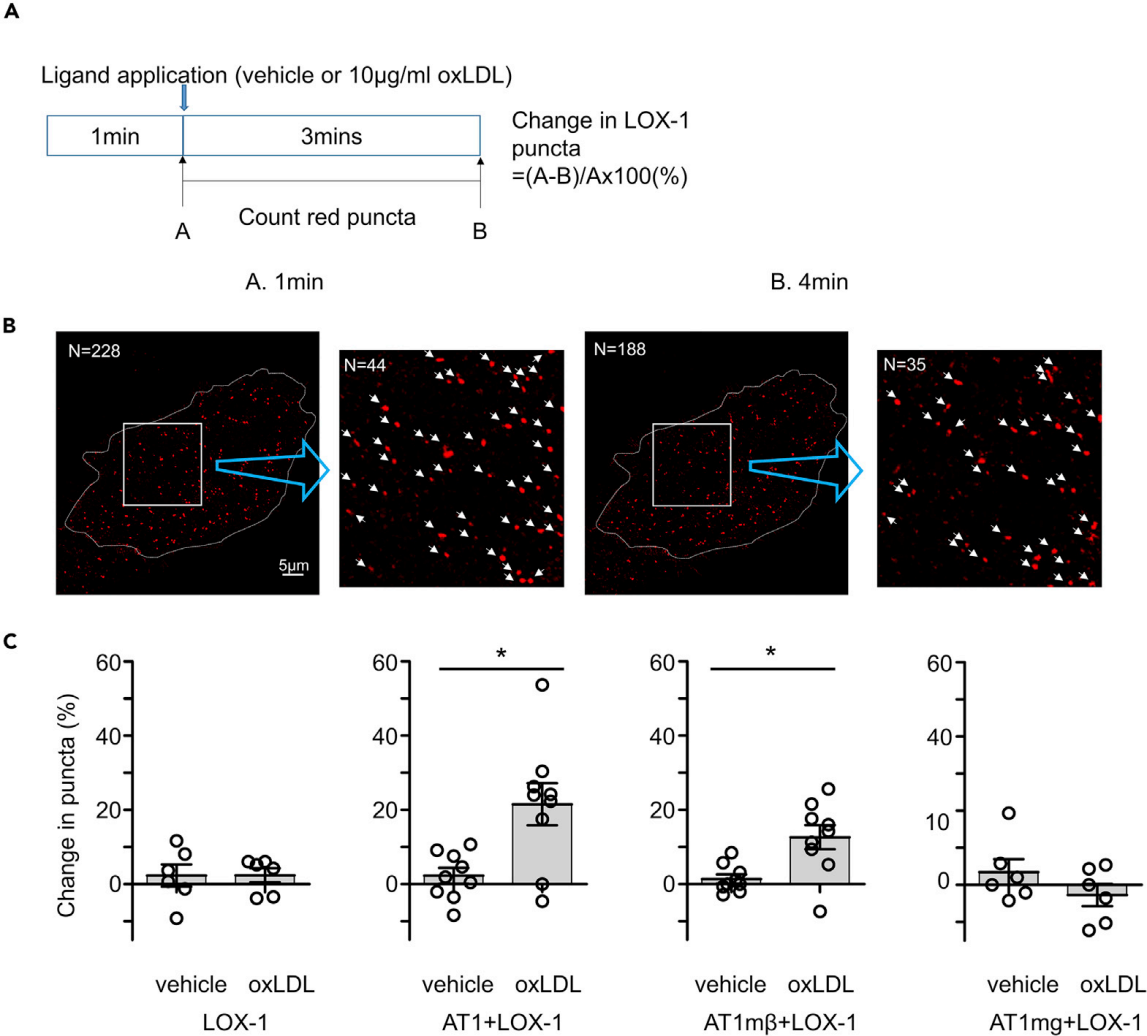
AT1-β-arrestin-dependent internalization of membrane LOX-1 in response to oxLDL treatment (A) Calculation of change in red puncta of LOX-1. Real-time membrane imaging of CHO-K1 cells co-transfected with LOX-1-mScarlet and control, AT1, AT1mβ, or AT1mg labeled with eGFP (Video S1). A count of puncta was performed using separated images visualizing LOX-1-scarlet just before and 3 min after ligand application. (B) Example of counting red puncta in CHO-K1 cells co-expressing LOX-1-scarlet and AT1-GFP applied with oxLDL. In this case, the change in LOX-1 puncta is (228 − 188)/288 × 100 = 17.5%. (C) Change in LOX-1 puncta in CHO-K1 cells expressing the indicated receptors (n = 6–9). Data are represented as mean ± SEM. The difference between the application of vehicle and oxLDL was determined by Student's t-test. ∗p < 0.01.

To investigate whether the activation of AT1 by oxLDL induced internalization of the LOX-1-AT1 complex, we utilized a live cell imaging technique using a real-time spinning disk confocal super-resolution microscope in CHO-K1 cells transiently transfected with LOX-1 labeled with mScarlet and AT1 labeled with eGFP. As shown in the Video S1, we observed yellow puncta, indicating co-localization of AT1 and LOX-1 moving on the cell surface-focused image, with some of them disappearing after application of 10 μg/mL oxLDL, implying that the complex of LOX-1 and AT1 had been internalized. To quantify the internalization of LOX-1 in response to oxLDL stimulation, we analyzed the percentage of red LOX-1 puncta that vanished during treatment with vehicle or 10 μg/mL oxLDL, by comparing images before and 3 min after application of each reagent in CHO cells expressing fluorescent-labeled receptors (Figures 4A and 4B). We found that oxLDL increased the number of vanished puncta in cells transfected with LOX-1 and AT1 but not in cells transfected with LOX-1 alone (Figure 4C). This phenomenon appeared to be β-arrestin dependent, as the increase in vanished LOX-1 puncta was similarly observed in response to oxLDL in cells transfected with LOX-1 and AT1mβ, but was totally dampened in cells transfected with LOX-1 and AT1mg (Figure 4C).

Video S1. Complete picture of membrane imaging of CHO-K1 cells co-transfected with LOX-1-scarlet and AT1-eGFP (left), related to Figure 4 Real-time membrane imaging of the selected area of the image at higher resolution (right).

### Intracellular accumulation of oxLDL depends on the concurrent presence of LOX-1 and AT1

**Figure 5:**
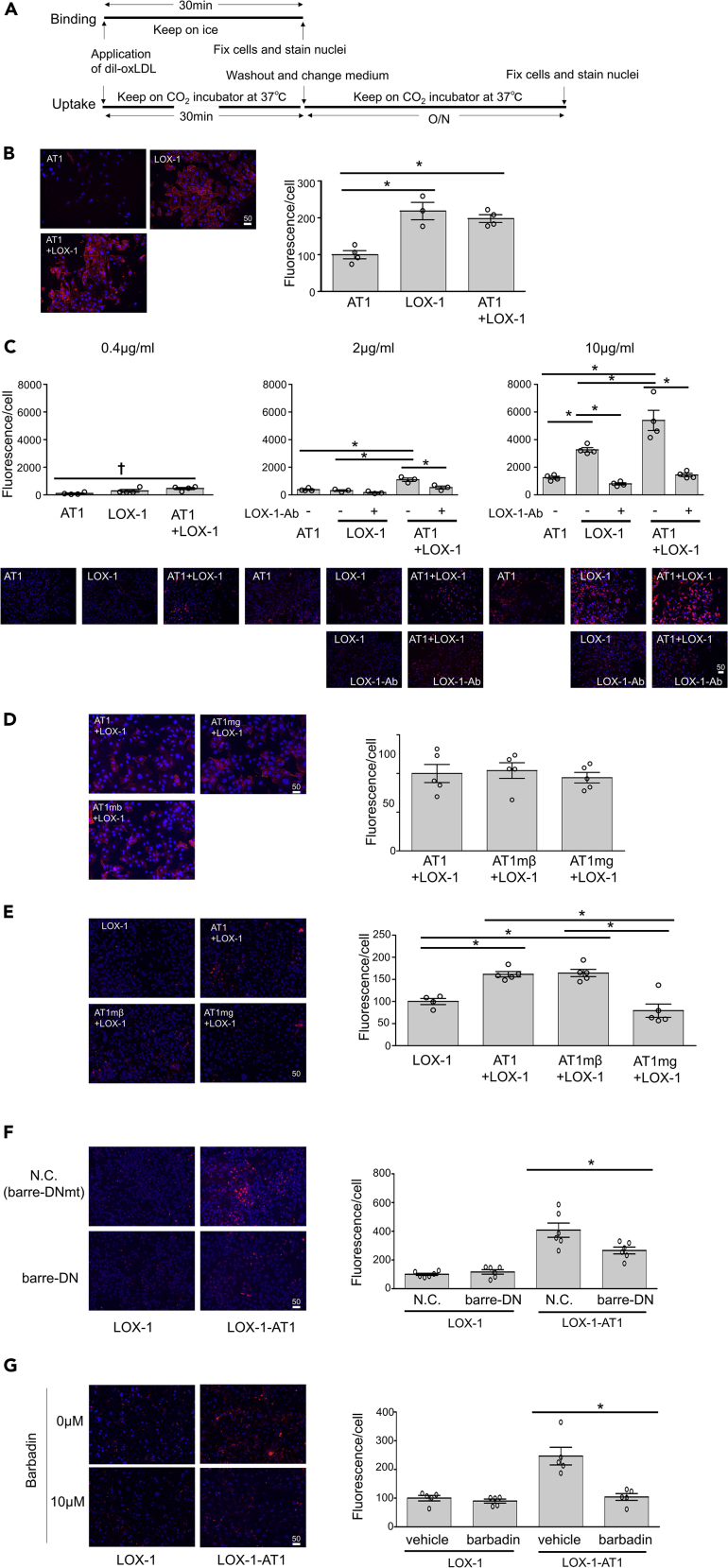
LOX-1-AT1-β-arrestin-dependent uptake of oxLDL in genetically engineered CHO cells (A) Protocol of visualization of membrane binding and intracellular uptake of Dil-labeled oxLDL in genetically engineered CHO cells. (B) Binding of 2 μg/mL Dil-labeled oxLDL in the indicated genetically engineered CHO cells (n = 4, each). Scale bar, μm. The graph indicates the fluorescence/number of nuclei, and the average value of the group at the far left was normalized to 100%. Data are represented as mean ± SEM. The differences were determined by one-way analysis of variance (ANOVA) with Bonferroni correction. ^∗^p < 0.01. (C) Intracellular uptake of the indicated concentration of Dil-labeled oxLDL in the indicated genetically engineered CHO cells. LOX-1 neutralizing antibody, TS92, was used to pretreat the cells at 10 μg/mL for 30 min before stimulation (n = 4, each). Scale bar, μm. The graph indicates the fluorescence/number of nuclei, and the average value of the group at the far left was normalized to 100%. Data are represented as mean ± SEM. The differences were determined by one-way ANOVA with Bonferroni correction. ^∗^p < 0.01, ^†^p < 0.05. (D) Binding of 2 μg/mL Dil-labeled oxLDL in the indicated genetically engineered CHO cells (n = 5, each). Scale bar, μm. The graph indicates the fluorescence/number of nuclei, and the average value of the group at the far left was normalized to 100%. Data are represented as mean ± SEM. The differences were determined by one-way ANOVA. (E) Intracellular uptake of 2 μg/mL Dil-labeled oxLDL in the indicated genetically engineered CHO cells (n = 5, each). Scale bar, μm. The graph indicates the fluorescence/number of nuclei, and the average value of the group at the far left was normalized to 100%. Data are represented as mean ± SEM. The differences were determined by one-way ANOVA with Bonferroni correction. ^∗^p < 0.01. (F) Intracellular uptake of 2 μg/mL Dil-labeled oxLDL in the indicated genetically engineered CHO cells transfected with dominant negative vector of β-arrestin (barre-DN) or negative control vector (N.C.) (n = 5, each). The vectors were transfected 24 h before stimulation with Dil-labeled oxLDL. Schematics of barre-DN and N.C. are shown in Figure 3 (E). Scale bar, μm. The graph indicates the fluorescence/number of nuclei, and the average value of the group at the far left was normalized to 100%. Data are represented as mean ± SEM. The differences were determined by one-way ANOVA with Bonferroni correction. ^∗^p < 0.01. (G) Intracellular uptake of 2 μg/mL Dil-labeled oxLDL in the indicated genetically engineered CHO cells pretreated with vehicle or the β-arrestin-specific inhibitor barbadin at 10 μM for 30 min (n = 5, each). Scale bar, μm. The graph indicates the fluorescence/number of nuclei, and the average value of the group at the far left was normalized to 100%. Data are represented as mean ± SEM. The differences were determined by one-way ANOVA with Bonferroni correction. ^∗^p < 0.01.

We used fluorescent oxLDL (Dil-oxLDL) to monitor cellular accumulation of oxLDL as a consequence of LOX-1 internalization. The binding of oxLDL to the cellular membrane was monitored after 30 min incubation with Dil-oxLDL at 4°C (Figure 5A). As shown in Figure 5B, oxLDL binds to LOX-1 at the cellular membrane in a similar manner between CHO-LOX-1 and CHO-LOX-1-AT1. Endocytic traffic of oxLDL was detected in CHO-LOX-1-AT1, where co-localization of oxLDL with early and late endosomes and lysosomes was observed, and the co-localization with lysosomes increased over time following 30 min of Dil-oxLDL treatment (Figure S1). Cellular accumulation of oxLDL was monitored in CHO cells after application of Dil-oxLDL for 30 min followed by washing of membrane-bound oxLDL for 24 h at 37°C (Figure 5A). Cellular accumulation of Dil-oxLDL was competitively blocked by co-treatment with non-fluorescent oxLDL, suggesting that the accumulation is not induced by the non-specific accumulation of Dil fluorescent dye (Figure S2). We found that cellular (fluorescent) oxLDL content was consistently higher in CHO-LOX-1-AT1 than in CHO-AT1 and CHO-LOX-1 after application of 0.4, 2, and 10 μg/mL oxLDL (Figure 5C). While cellular oxLDL content was similar between CHO-AT1 and CHO-LOX-1 after the application of 2 μg/mL oxLDL, 10 μg/mL oxLDL accumulated more vigorously in CHO-LOX-1 than in CHO-AT1, suggesting the presence of AT1-independent uptake of oxLDL via LOX-1 (Figure 5C). Pretreatment with the LOX-1 antibody dampened the increased uptake of oxLDL in the LOX-1-expressing CHO cells (Figure 5C).

### Intracellular accumulation of oxLDL in the presence of LOX-1 is stimulated by the activation of a β-arrestin-dependent signaling pathway of AT1

We found that oxLDL binding to LOX-1 on the cellular membrane was similarly observed in CHO-LOX-1-AT1, CHO-LOX-1-AT1mβ, and CHO-LOX-1-AT1mg cells (Figure 5D). As shown in Figure 5E, the cellular oxLDL content after the application of 2 μg/mL Dil-oxLDL was higher in CHO-LOX-1-AT1 and CHO-LOX-1-AT1mβ than in CHO-LOX-1-AT1mg and CHO-LOX-1, suggesting that the uptake of oxLDL through AT1 was β-arrestin dependent (Figure 5E). The mechanism was further confirmed using transfection of dominant negative (DN) β-arrestin (Krupnick et al., 1997) as compared to that of the negative control vector harboring mutated DN-β-arrestin, in which the clathrin-binding domain was deleted (Kang et al., 2009), or barbadin, an inhibitor of the β-arrestin/AP2 endocytic complex (Beautrait et al., 2017) (Figures 5F and 5G). Transfection of DN β-arrestin decreased the uptake of 2 μg/mL oxLDL in CHO-LOX-1-AT1 compared to that in cells transfected with the negative control vector, while no difference was observed in similarly treated CHO-LOX-1, further supporting the β-arrestin-dependent uptake of oxLDL through AT1 (Figure 5F). A similar effect of DN-β-arrestin was observed upon treatment with 10 μg/mL oxLDL in CHO-LOX-1-AT1 but not CHO-LOX-1, suggesting that the AT1-independent uptake of a higher concentration of oxLDL via LOX-1 (Figure 5C) was β-arrestin independent (Figure S3). The β-arrestin-induced endocytosis of oxLDL was also supported by barbadin, which inhibits the accumulation of oxLDL in CHO-LOX-1-AT1 but not in CHO-LOX-1 (Figure 5G). We confirmed that the cellular accumulation of oxLDL does not depend on Gαi or Gαq-dependent pathways, as pharmacological inhibitors of Gαq and Gαi had no effect on oxLDL accumulation in CHO-LOX-1-AT1 (Figure S4).

### LOX-1-AT1-β-arrestin-dependent oxLDL uptake is relevant in human vascular endothelial cells

**Figure 6:**
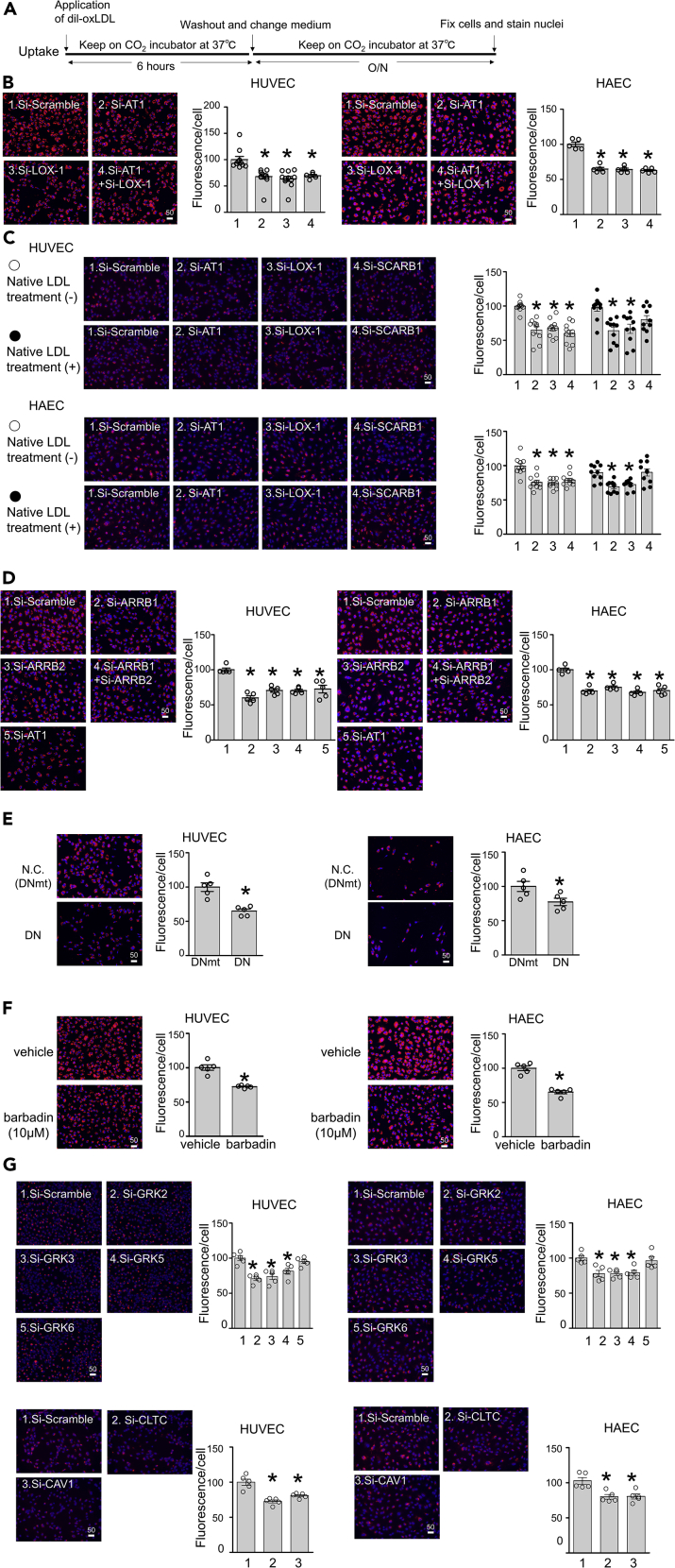
LOX-1-AT1-β-arrestin-dependent uptake of oxLDL in human endothelial cells (A) Protocol for visualization of intracellular uptake of Dil-labeled oxLDL in human umbilical vein endothelial cells (HUVECs) and human aortic endothelial cells (HAECs). (B) Intracellular uptake of 2 μg/mL Dil-labeled oxLDL in cells with siRNA knockdown of indicated genes (n = 5, each). Scale bar, μm. The graph indicates the fluorescence/number of nuclei, and the average value of the group at the far left was normalized to 100%. Data are represented as mean ± SEM. The differences were determined by one-way analysis of variance (ANOVA) with Bonferroni correction. ^∗^p < 0.01 vs. control. The knockdown efficiency of each gene was demonstrated in Figure S6. (C) Intracellular uptake of 2 μg/mL Dil-labeled oxLDL in cells with siRNA-mediated knockdown of the indicated genes, pre- and co-treated or not with non-labeled native LDL at 20 μg/mL (n = 10, each). Pretreatment with native LDL was carried out for 1 hr. Scale bar, μm. The graph indicates the fluorescence/number of nuclei, and the average value of the group at the far left was normalized to 100%. Data are represented as mean ± SEM. The differences were determined by one-way ANOVA with Bonferroni correction. ^∗^p < 0.01 vs. control. The knockdown efficiency of each gene was demonstrated in Figure S6. (D) Intracellular uptake of 2 μg/mL Dil-labeled oxLDL in cells with siRNA-mediated knockdown of the indicated genes (n = 5, each). Scale bar, μm. The graph indicates the fluorescence/number of nuclei, and the average value of the group at the far left was normalized to 100%. Data are represented as mean ± SEM. The differences were determined by one-way ANOVA with Bonferroni correction. ^∗^p < 0.01 vs. control. The knockdown efficiency of each gene was demonstrated in Figure S6. (E) Intracellular uptake of 2 μg/mL Dil-labeled oxLDL in cells transfected with dominant negative vector of β-arrestin (barre-DN) or negative control vector (N.C.) (n = 5, each). Scale bar, μm. The graph indicates the fluorescence/number of nuclei, and the average value of the group at the far left was normalized to 100%. Data are represented as mean ± SEM. The differences were determined by Student's t-test. ^∗^p < 0.01 vs. control (DNmt). (F) Intracellular uptake of 2 μg/mL Dil-labeled oxLDL in cells pretreated with vehicle or the β-arrestin-specific inhibitor barbadin at 10 μM for 30 min (n = 5, each). The graph indicates the fluorescence/number of nuclei, and the average value of the group at the far left was normalized to 100%. Scale bar, μm. The graph indicates the fluorescence/number of nuclei, and the average value of the group at the far left was normalized to 100%. Data are represented as mean ± SEM. The differences were determined by Student's t-test. ^∗^p < 0.01 vs. control. (G and H) Intracellular uptake of 2 μg/mL Dil-labeled oxLDL in cells with siRNA-mediated knockdown of the indicated genes (n = 5, each). Scale bar, μm. The graph indicates the fluorescence/number of nuclei, and the average value of the group at the far left was normalized to 100%. Data are represented as mean ± SEM. The differences were determined by one-way ANOVA with Bonferroni correction. ^∗^p < 0.01 vs. control. The knockdown efficiency of each gene was demonstrated in Figure S6.

We explored the biological relevance of the LOX-1-AT1-β-arrestin-dependent uptake of oxLDL in human vascular endothelial cells (Figure 6A). Similar to the findings in CHO-LOX-1-AT1 (Figure S1), endocytic traffic of oxLDL was detected in human umbilical vein endothelial cells (HUVECs). In these cells, co-localization of oxLDL with early and late endosomes and lysosomes was observed, which increased over time after 30 min of Dil-oxLDL treatment for late endosomes and lysosomes (Figure S5). We found that oxLDL accumulated in HUVECs and human aortic endothelial cells (HAECs) after the application of 2 μg/mL Dil-oxLDL for 6 h followed by washing for 24 h (Figure 6B). Genetic knockdown of *AT1* or *LOX-1* by small interfering RNA (siRNA) prior to Dil-oxLDL application similarly inhibited the accumulation of oxLDL in these cells (Figure 6B, knockdown efficiency shown in Figure S6A). Simultaneous knockdown of *AT1* and *LOX-1* had no additional effect compared to the knockdown of each gene alone, suggesting that both receptors share the same pathway for oxLDL uptake (Figure 6B). The inhibitory effect of siRNA against *AT1* or *LOX-1* was also observed in these cells, even after a quick Dil-oxLDL treatment (10 min or 30 min), suggesting the rapid kinetics of this phenomenon (Figure S7). It was recently reported that SR-B1 promotes transcytosis of both native LDL and oxLDL in endothelial cells (Huang et al., 2019). Consistently, we found that siRNA targeting *SCARB1*, the gene encoding SR-BI, inhibited the accumulation of Dil-oxLDL to a similar to siRNA targeting *AT1* or *LOX-1* (Figure 6C, knockdown efficiency shown in Figure S6B). In contrast, pre- and co-treatment with 20 μg/mL native LDL hampered this inhibitory effect of siRNA on *SCARB1*, whereas such treatments did not influence the effect of siRNA on *AT1* or *LOX-1* (Figure 6C). This result is consistent with the binding specificity of LOX-1 for modified LDL. We also found that knockdown of β-arrestin 1 (*ARRB1*) and/or β-arrestin 2 (*ARRB2*) inhibited the accumulation of oxLDL in HUVECs and HAECs to a similar extent as observed in cells with knockdown of *AT1* (Figure 6D). β-arrestin-dependent uptake of oxLDL in endothelial cells was further supported by DN or pharmacological inhibition of β-arrestin, both of which inhibited the uptake of oxLDL in HUVECs and HAECs (Figures 6E and 6F). β-arrestin is known to mediate G-protein-independent activation of extracellular signal-regulated kinase 1/2 by Ang II (Lee et al., 2008). In line with the literature, we found that the activation of ERK1/2 in HUVECs, triggered by a 10-min treatment with oxLDL, was inhibited, at least partially, by treatment with barbadin, while this phenomenon did not occur in presence of the Gαi inhibitor PTX, consistent with our previous study (Yamamoto et al., 2015) (Figure S8). We also found that the activation of ERK 1/2 by the co-treatment of 20μg/ml oxLDL with 10^−7^MAng II was equivalent to that by Ang II alone and lower than that by oxLDL alone in HUVECs, suggesting that the effect of Ang II and oxLDL on cellular signaling is not additive but rather competitive in certain experimental condition in endothelial cells (Figure S9).

### GPCR kinases and clathrin are involved in oxLDL uptake by human vascular endothelial cells

The interaction of GPCR kinases (GRKs) with the cytoplasmic tail of AT1 is a crucial step for Ang II-induced AT1 internalization, followed by β-arrestin recruitment (Grisanti et al., 2018; Sato et al., 2015). Therefore, we used siRNA-mediated knockdown of genes encoding the four major isoforms of GRKs (GRK2, GRK3, GRK5, and GRK6) to investigate the involvement of these proteins in the uptake of oxLDL by endothelial cells (Kim et al., 2005; Oppermann et al., 1996). We found that siRNA targeting *GRK2* or *GRK3* decreased gene expression each other (Figure S6C). siRNA targeting *GRK2*, *GRK3*, or *GRK5* inhibited oxLDL uptake in HUVECs and HAECs, whereas siRNA targeting *GRK6* had no effect on this process (Figure 6G, knockdown efficiency shown in Figure S6C). Although Ang II-induced AT1 internalization occurs via clathrin-dependent endocytosis, Murphy et al. reported the presence of LOX-1- and clathrin-independent mechanisms of oxLDL uptake (Murphy et al., 2008). Therefore, as caveolae-mediated endocytosis of oxLDL was observed in HUVECs (Sun et al., 2010), we tested whether the uptake of oxLDL in endothelial cells was affected by siRNA-mediated knockdown of *CHC17* or caveolin-1, encoding the key molecules for the formation of clathrin or caveolae, respectively. We found that siRNA targeting either *CHC17* or caveolin-1 inhibited the uptake of Dil-oxLDL in HUVECs and HAECs, suggesting the involvement of both clathrin-dependent and clathrin-independent pathways in the endocytosis of oxLDL (Figure 6H, knockdown efficiency shown in Figure S6D).

### Pharmacological blockade of AT1 has no effect on β-arrestin-dependent internalization of the oxLDL-LOX-1-AT1 complex

We previously reported that G-protein-dependent cell signaling of AT1 provoked by the binding of oxLDL to LOX-1 is inhibited by treatment with AT1 blockers (ARBs) that competitively bind to the Ang II-binding pocket of AT1 (Yamamoto et al., 2015). Finally, we tested whether ARBs could inhibit the β-arrestin-dependent internalization of the oxLDL-LOX-1-AT1 complex. As a result, pretreatment with a high concentration (10 μM) of ARBs, losartan, telmisartan, or irbesartan had no effect on oxLDL uptake in CHO-LOX-1-AT1 or HUVECs (Figure S10).

## Discussion

In the current study, we found that the activation of AT1 by the binding of oxLDL to LOX-1 is distinct from the activation of AT1 by its orthosteric ligand Ang II, in terms of its selectivity in G protein signaling and the biological significance of β-arrestin-dependent receptor internalization. Given that Ang II elicits its pressor action primarily by Gq-dependent activation of AT1, the absence of AT1-Gq activation by oxLDL can answer the question raised in our previous study (Yamamoto et al., 2015) regarding the lack of association between hypertension and elevated circulating oxLDL concentration in patients with atherosclerosis (Toshima et al., 2000).

Clathrin-mediated endocytosis of AT1 via a β-arrestin-dependent pathway is widely recognized as a cellular desensitization process by which the response to repetitive stimulation of Ang II is attenuated (Turu et al., 2006; Violin and Lefkowitz, 2007). Our current findings strongly suggest that oxLDL triggers the activation of GRKs, followed by β-arrestin-induced clathrin-dependent endocytosis of the AT1-LOX-1 complex, whereby circulating oxLDL is translocated into vascular endothelial cells. The uptake of oxLDL in the vascular endothelium by LOX-1 is involved in the development of atherosclerosis (Kita et al., 2001; Li and Mehta, 2005; Mehta et al., 2006). Our findings raise the possibility that AT1-mediated internalization of oxLDL and LOX-1 may be of pathophysiological significance, leading to a conceptual shift in understanding of the functional significance of AT1 internalization beyond its role in receptor desensitization. Taken together, these findings explain the difference in physiological and pathophysiological consequences of AT1 activation induced by oxLDL and Ang II.

AT1-induced activation of β-arrestin has been conventionally detected using a bioluminescent resonance energy transfer (BRET) assay. In the present study, we attempted to detect the oxLDL-induced BRET between AT1-Rluc8 and β-arrestin 2 (ARRB2)-mVenus in CHO-K1 cells in the presence of LOX-1. However, this trial failed because the oxLDL solution substantially attenuated the luminescence, resulting in oxLDL-induced elevation of the BRET ratio even in the absence of β-arrestin (ARRB)-mVenus (Figure S11). Instead, the signatures of LOX-1-AT1-dependent β-arrestin activation were observed by the detection of the molecular dynamics of LOX-1 on the cellular membrane in high-resolution live cell imaging (Figures 4A–4C). Given the observation that some merged puncta of AT1 and LOX-1 disappeared after the application of oxLDL, we conducted a quantitative analysis to detect intracellular trafficking of LOX-1 in response to oxLDL. While time-dependent changes in LOX-1 puncta could be a reflection of both its appearance and disappearance from the cellular membrane, a substantial decline in LOX-1 puncta in response to oxLDL was observed in parallel with the ability of AT1 to activate arrestin signaling.

Finally, endocytotic internalization of oxLDL was shown by the sublocalization of oxLDL through endosomes to lysosomes (Figures S1 and 5). This pathway was AT1-arrestin dependent as shown by microscopy analysis in which the cellular contents of fluorescent oxLDL were prominently attenuated by the ablation of the arrestin pathway of AT1 (Figure 4). Altogether, these findings establish a transport mechanism whereby the oxLDL-LOX-1-AT1 complex is internalized in cells via the β-arrestin-dependent endocytosis of AT1. It should be noted that LOX-1-dependent uptake of oxLDL involved an AT1-arrestin-independent pathway, as higher concentrations of oxLDL (10 μg/mL) increased the cellular content of oxLDL in CHO-LOX-1, which was not altered by DN β-arrestin (Figures 5C and S3). This finding is consistent with those of a previous study showing that LOX-1 alone or oxLDL binding to LOX-1 (10 μg/mL) undergoes ligand-independent constitutive internalization depending on its cytoplasmic tripeptide motif (Murphy et al., 2008). Indeed, 10 μg/mL oxLDL did not alter the dynamics of membrane LOX-1 in cells overexpressing LOX-1 alone, suggesting that the internalization of LOX-1 at this concentration involves a ligand-independent pathway (Figure 4). The observation of this LOX-1- and AT1-independent endocytic pathway is consistent with the study by Murphy et al. (2008), in which siRNA targeting *CHC17* did not inhibit the uptake of oxLDL in HeLa cells overexpressing LOX-1 alone. The existence of clathrin-independent endocytosis was also supported by the finding that loss of function of caveolae or clathrin inhibited the uptake of oxLDL in endothelial cells (Figure 6H). Nevertheless, it is conceivable that the AT1-independent endocytosis of the LOX-1-oxLDL complex is additive but not competitive with AT1-dependent endocytosis, as uptake of 10 μg/mL oxLDL was higher in CHO-LOX-1-AT1 than in CHO-LOX-1 (Figure 5C).

The question here is how oxLDL binding to LOX-1 stimulates such biased activation of AT1. AT1 forms a heterodimer with other GPCRs, and heterodimers tend to change the G protein preference and β-arrestin binding affinity (Barki-Harrington et al., 2003; Bellot et al., 2015; Nishimura et al., 2016; Quitterer et al., 2019; Rozenfeld et al., 2011; Siddiquee et al., 2013). However, while the known heterodimers of AT1 affect the signaling patterns of AT1 activation induced by its orthosteric ligand Ang II, there is no precedence of our finding that a receptor utilizes the adjacent GPCR as a mediator of signal transduction and a transporter of its ligand. Nevertheless, some clues for potential mechanisms have been provided by recent advances in characterizing biased AT1 activation by various stimuli. SII is a well-known biased agonist of AT1 that selectively activates β-arrestin, but recent findings have revealed its potential to activate G protein as well (Domazet et al., 2015; Sauliere et al., 2012). More recently, various Ang II analogs, including SII, were tested for the selectivity of G protein activation and β-arrestin signaling, and most of the analogs induced bias responses of AT1 against Gαq in favor of Gαi, Gα12, and β-arrestin (Namkung et al., 2018). AT1 is activated by mechanical stretch (Zou et al., 2004), and this activation is selective (Rakesh et al., 2010; Tang et al., 2014). Interestingly, Wang et al. (2018) recently reported that the mechanical stretch of AT1 activates Gαi but not Gq, which then mediates β-arrestin biased signaling. This Gαi-dependent activation of β-arrestin signaling is distinct from the current finding that β-arrestin signaling by oxLDL is not altered by the impaired ability of AT1 to activate G protein. However, the preference to activate Gαi compared to Gq appears to be consistent among various allosteric stimulators of AT1. Previous studies have suggested that the distinct signaling pathway of AT1 induced by different ligands depends on the ligand-specific structural change of the receptor. There are four crystal structures for inactive or active forms of AT1 reported in the Protein Data Bank (Clement et al., 2005; Quitterer et al., 2019; Zhang et al., 2015a, 2015b). Interestingly, recent structural analysis using double electron-electron resonance spectroscopy indicated that β-arrestin biased agonists induce less “open’’ conformational changes in AT1 than Ang II or agonists with enhanced Gαq coupling (Wingler et al., 2019). Further investigation is required to reveal the structural switch of AT1 induced by oxLDL binding to LOX-1, leading to a similar biased signaling pathway prevalent across diverse stimuli.

Interestingly, we found that pharmacological inhibition of AT1 by ARBs did not alter the β-arrestin-dependent internalization of the oxLDL-LOX-1-AT1 complex. This is in contrast to our previous findings that G-protein-dependent signaling of oxLDL-induced AT1 activation is inhibited by ARBs (Yamamoto et al., 2015). The concept of ARBs to inhibit oxLDL-LOX-1-induced AT1 activation is theoretically similar to that of “inverse agonist” potential of ARBs to inhibit constitutive activation of AT1 induced by mechanical stretch (Takezako et al., 2015). It is conceivable that the impact of ARBs on the oxLDL-LOX-1-induced structural change of AT1 is sufficient to block the G-protein-dependent pathway but insufficient to block the β-arrestin-dependent pathway. Further investigation is required to clarify the structural basis of the selectivity of ARBs in inhibiting oxLDL-induced AT1 activation.

We have demonstrated that a mechanism involving AT1 and β-arrestin can influence the uptake of oxLDL in human vascular endothelial cells; however, the pathophysiological significance of this phenomenon remains to be fully determined. We found that co-localization of oxLDL with lysosomes increased over time after endocytosis in cells expressing both AT1 and LOX-1, suggesting that the majority of oxLDL is terminally cleared from cells (Figures S1 and 5). This pathway is clearly distinct from the transcytosis of oxLDL, which was recently found to depend on SR-BI (Figure 6C) (Huang et al., 2019). We previously reported that oxLDL-induced endothelial dysfunction of the aortic ring was inhibited by ARB, as well as genetic deletion of *AT1a* in mice (Yamamoto et al., 2015). Therefore, although we found that the ERK1/2 activation induced by oxLDL was partially blocked upon inhibition of β-arrestin (Figure S8), it is conceivable that β-arrestin-dependent accumulation of oxLDL does not primarily contribute to endothelial dysfunction in normal tissues. Indeed, we found that the oxLDL-induced inflammatory response detected by the activation of NfκB depended on G protein signaling but not on β-arrestin signaling of AT1 (Figure 3A). Nevertheless, recent studies have suggested that the disruption of the endothelial autophagy-lysosomal pathway enhances the development of atherosclerosis, and oxLDL induces lysosomal dysfunction (Emanuel et al., 2014; Torisu et al., 2016; Vion et al., 2017). Further investigation is required to elucidate how far the endothelial uptake of oxLDL via the AT1-LOX-1 pathway contributes to the development of atherosclerosis *in vivo*. Additionally, LOX-1 belongs to the c-type lectin receptor family and functions as a pattern recognition receptor (PRR) that binds to multiple ligands, primarily including damage-associated molecular patterns and pathogen-associated molecular patterns (Miller et al., 2011). LOX-1 binds to multiple ligands besides oxLDL, including C-reactive protein, modified high-density lipoprotein, and remnant lipoprotein, all of which could promote atherosclerosis (Besler et al., 2011; Fujita et al., 2009; Shin et al., 2004).

Local production of Ang II may also promote atherosclerosis (Brasier et al., 2002). It has been shown that the development of atherosclerosis is attenuated by the genetic deletion of either AT1 (Daugherty et al., 2004; Wassmann et al., 2004) or LOX-1 (Mehta et al., 2007) in atherogenic mice. However, since our findings imply that multiple molecules share the same LOX-1-AT1 system to exert their roles in atherogenesis, it is difficult to determine how these individual molecules utilize the system and, thus, contribute to disease progression. In addition, it is of interest to clarify which pathways are more important in the development of atherosclerosis: G-protein-dependent induction of vascular damage or β-arrestin-dependent vascular accumulation of LOX-1 ligands. This is important from a therapeutic point of view, given the inhibitory effect of ARBs in the AT1-G protein-dependent pathway but not in the β-arrestin-dependent pathway. It also remains to be determined whether these distinct molecular pathways can synergistically contribute to the development of atherosclerosis. Finally, it is important to elucidate whether the canonical Ang II signaling pathway is affected by oxLDL-induced AT1 activation, and vice versa, because Ang II and oxLDL utilize the same receptor to trigger biological reactions *in vivo*. Indeed, we found the contrast cellular response by the combined treatment of Ang II with oxLDL in different assays and cell types. While we found that the combined treatment of Ang II and oxLDL prominently enhanced NfκB activity in CHO-LOX-1-AT1 (Figure 3B), the combined treatment of these ligands did not enhance the activation of ERK 1/2 compared to each treatment alone in endothelial cells (Figure S9). These results suggest that the combination of Ang II-AT1 signaling and oxLDL-LOX-1-AT1 signaling could exert either inhibitory or additive cellular response depending on undefined factors. The net cellular reaction in a milieu with the co-presence of Ang II and oxLDL can be influenced by many factors including the concentration of these ligands, the abundance of the receptors, and cell types. Further investigation using specific animal models is required to clarify these questions and understand the overall pathophysiological relevance of this molecular machinery.

In conclusion, we demonstrated the first example of a transport system whereby a PRR ligand stimulates biased signaling pathways of GPCR, which consequently leads to intracellular trafficking of the ligand. Given the ability of PRRs to capture diverse ligands and their physiological and pathophysiological significance, it will be of interest to determine the extent to which our findings regarding LOX-1 pertain to other PRRs and to evaluate the importance of these findings in health and disease.

### Limitations of the study

Here, we use CHO cells, HUVECs, and HAECs. We have not yet analyzed the pathophysiological mechanisms *in vivo*.

### Resource availability

#### Lead contact

Further information and requests for resources and reagents should be directed to and will be fulfilled by the lead contact, Koichi Yamamoto kyamamoto@geriat.med.osaka-u.ac.jp.

#### Material availability

This study did not include any new unique reagents, and all reagents generated in this study are available from the lead contact without restriction.

#### Data and code availability

The data sets supporting the current study are available from the lead contact upon reasonable request.

## Methods

All methods can be found in the accompanying Transparent methods supplemental file.

## Supplemental information

Document S1. Transparent methods and Figures S1–S11

## References

[bib1] Barki-Harrington L., Luttrell L.M., Rockman H.A. (2003). Dual inhibition of beta-adrenergic and angiotensin II receptors by a single antagonist: a functional role for receptor-receptor interaction in vivo. Circulation.

[bib2] Beautrait A., Paradis J.S., Zimmerman B., Giubilaro J., Nikolajev L., Armando S., Kobayashi H., Yamani L., Namkung Y., Heydenreich F.M. (2017). A new inhibitor of the beta-arrestin/AP2 endocytic complex reveals interplay between GPCR internalization and signalling. Nat. Commun..

[bib3] Bellot M., Galandrin S., Boularan C., Matthies H.J., Despas F., Denis C., Javitch J., Mazeres S., Sanni S.J., Pons V. (2015). Dual agonist occupancy of AT1-R-alpha2C-AR heterodimers results in atypical Gs-PKA signaling. Nat. Chem. Biol..

[bib4] Berk B.C. (1999). Angiotensin II signal transduction in vascular smooth muscle: pathways activated by specific tyrosine kinases. J. Am. Soc. Nephrol..

[bib5] Besler C., Heinrich K., Rohrer L., Doerries C., Riwanto M., Shih D.M., Chroni A., Yonekawa K., Stein S., Schaefer N. (2011). Mechanisms underlying adverse effects of HDL on eNOS-activating pathways in patients with coronary artery disease. J. Clin. Invest..

[bib6] Brasier A.R., Recinos A., Eledrisi M.S. (2002). Vascular inflammation and the renin-angiotensin system. Arterioscler Thromb. Vasc. Biol..

[bib7] Clement M., Martin S.S., Beaulieu M.E., Chamberland C., Lavigne P., Leduc R., Guillemette G., Escher E. (2005). Determining the environment of the ligand binding pocket of the human angiotensin II type I (hAT1) receptor using the methionine proximity assay. J. Biol. Chem..

[bib8] Daugherty A., Rateri D.L., Lu H., Inagami T., Cassis L.A. (2004). Hypercholesterolemia stimulates angiotensin peptide synthesis and contributes to atherosclerosis through the AT1A receptor. Circulation.

[bib9] Domazet I., Holleran B.J., Richard A., Vandenberghe C., Lavigne P., Escher E., Leduc R., Guillemette G. (2015). Characterization of angiotensin II molecular determinants involved in AT1 receptor functional selectivity. Mol. Pharmacol..

[bib10] Emanuel R., Sergin I., Bhattacharya S., Turner J., Epelman S., Settembre C., Diwan A., Ballabio A., Razani B. (2014). Induction of lysosomal biogenesis in atherosclerotic macrophages can rescue lipid-induced lysosomal dysfunction and downstream sequelae. Arterioscler Thromb. Vasc. Biol..

[bib11] Ferre S., Casado V., Devi L.A., Filizola M., Jockers R., Lohse M.J., Milligan G., Pin J.P., Guitart X. (2014). G protein-coupled receptor oligomerization revisited: functional and pharmacological perspectives. Pharmacol. Rev..

[bib12] Forrester S.J., Booz G.W., Sigmund C.D., Coffman T.M., Kawai T., Rizzo V., Scalia R., Eguchi S. (2018). Angiotensin II signal transduction: an update on mechanisms of physiology and pathophysiology. Physiol. Rev..

[bib13] Fujita Y., Kakino A., Nishimichi N., Yamaguchi S., Sato Y., Machida S., Cominacini L., Delneste Y., Matsuda H., Sawamura T. (2009). Oxidized LDL receptor LOX-1 binds to C-reactive protein and mediates its vascular effects. Clin. Chem..

[bib14] Gentry P.R., Sexton P.M., Christopoulos A. (2015). Novel allosteric modulators of G protein-coupled receptors. J. Biol. Chem..

[bib15] Grisanti L.A., Schumacher S.M., Tilley D.G., Koch W.J. (2018). Designer approaches for G protein-coupled receptor modulation for cardiovascular disease. JACC Basic Transl Sci..

[bib16] Haendeler J., Ishida M., Hunyady L., Berk B.C. (2000). The third cytoplasmic loop of the angiotensin II type 1 receptor exerts differential effects on extracellular signal-regulated kinase (ERK1/ERK2) and apoptosis via Ras- and Rap1-dependent pathways. Circ. Res..

[bib17] Hermosilla T., Encina M., Morales D., Moreno C., Conejeros C., Alfaro-Valdes H.M., Lagos-Meza F., Simon F., Altier C., Varela D. (2017). Prolonged AT1R activation induces CaV1.2 channel internalization in rat cardiomyocytes. Sci. Rep..

[bib18] Huang L., Chambliss K.L., Gao X., Yuhanna I.S., Behling-Kelly E., Bergaya S., Ahmed M., Michaely P., Luby-Phelps K., Darehshouri A. (2019). SR-B1 drives endothelial cell LDL transcytosis via DOCK4 to promote atherosclerosis. Nature.

[bib19] Hunyady L. (1999). Molecular mechanisms of angiotensin II receptor internalization. J. Am. Soc. Nephrol..

[bib20] Hunyady L., Catt K.J. (2006). Pleiotropic AT1 receptor signaling pathways mediating physiological and pathogenic actions of angiotensin II. Mol. Endocrinol..

[bib21] Hunyady L., Catt K.J., Clark A.J., Gaborik Z. (2000). Mechanisms and functions of AT(1) angiotensin receptor internalization. Regul. Pept..

[bib22] Jean-Charles P.Y., Kaur S., Shenoy S.K. (2017). G protein-coupled receptor signaling through beta-arrestin-dependent mechanisms. J. Cardiovasc. Pharmacol..

[bib23] Kang D.S., Kern R.C., Puthenveedu M.A., von Zastrow M., Williams J.C., Benovic J.L. (2009). Structure of an arrestin2-clathrin complex reveals a novel clathrin binding domain that modulates receptor trafficking. J. Biol. Chem..

[bib24] Khoury E., Clement S., Laporte S.A. (2014). Allosteric and biased g protein-coupled receptor signaling regulation: potentials for new therapeutics. Front. Endocrinol. (Lausanne).

[bib25] Kim J., Ahn S., Ren X.R., Whalen E.J., Reiter E., Wei H., Lefkowitz R.J. (2005). Functional antagonism of different G protein-coupled receptor kinases for beta-arrestin-mediated angiotensin II receptor signaling. Proc. Natl. Acad. Sci. U S A..

[bib26] Kita T., Kume N., Minami M., Hayashida K., Murayama T., Sano H., Moriwaki H., Kataoka H., Nishi E., Horiuchi H. (2001). Role of oxidized LDL in atherosclerosis. Ann. N. Y Acad. Sci..

[bib27] Krupnick J.G., Santini F., Gagnon A.W., Keen J.H., Benovic J.L. (1997). Modulation of the arrestin-clathrin interaction in cells. Characterization of beta-arrestin dominant-negative mutants. J. Biol. Chem..

[bib28] Lee M.H., El-Shewy H.M., Luttrell D.K., Luttrell L.M. (2008). Role of beta-arrestin-mediated desensitization and signaling in the control of angiotensin AT1a receptor-stimulated transcription. J. Biol. Chem..

[bib29] Li D., Mehta J.L. (2005). Oxidized LDL, a critical factor in atherogenesis. Cardiovasc. Res..

[bib30] Mehta J.L., Chen J., Hermonat P.L., Romeo F., Novelli G. (2006). Lectin-like, oxidized low-density lipoprotein receptor-1 (LOX-1): a critical player in the development of atherosclerosis and related disorders. Cardiovasc. Res..

[bib31] Mehta J.L., Sanada N., Hu C.P., Chen J., Dandapat A., Sugawara F., Satoh H., Inoue K., Kawase Y., Jishage K. (2007). Deletion of LOX-1 reduces atherogenesis in LDLR knockout mice fed high cholesterol diet. Circ. Res..

[bib32] Miller Y.I., Choi S.H., Wiesner P., Fang L., Harkewicz R., Hartvigsen K., Boullier A., Gonen A., Diehl C.J., Que X. (2011). Oxidation-specific epitopes are danger-associated molecular patterns recognized by pattern recognition receptors of innate immunity. Circ. Res..

[bib33] Murphy J.E., Vohra R.S., Dunn S., Holloway Z.G., Monaco A.P., Homer-Vanniasinkam S., Walker J.H., Ponnambalam S. (2008). Oxidised LDL internalisation by the LOX-1 scavenger receptor is dependent on a novel cytoplasmic motif and is regulated by dynamin-2. J. Cell Sci..

[bib34] Namkung Y., LeGouill C., Kumar S., Cao Y., Teixeira L.B., Lukasheva V., Giubilaro J., Simoes S.C., Longpre J.M., Devost D. (2018). Functional selectivity profiling of the angiotensin II type 1 receptor using pathway-wide BRET signaling sensors. Sci. Signal..

[bib35] Nishimura A., Sunggip C., Tozaki-Saitoh H., Shimauchi T., Numaga-Tomita T., Hirano K., Ide T., Boeynaems J.M., Kurose H., Tsuda M. (2016). Purinergic P2Y6 receptors heterodimerize with angiotensin AT1 receptors to promote angiotensin II-induced hypertension. Sci. Signal..

[bib36] Oppermann M., Freedman N.J., Alexander R.W., Lefkowitz R.J. (1996). Phosphorylation of the type 1A angiotensin II receptor by G protein-coupled receptor kinases and protein kinase C. J. Biol. Chem..

[bib37] Qian H., Pipolo L., Thomas W.G. (2001). Association of beta-Arrestin 1 with the type 1A angiotensin II receptor involves phosphorylation of the receptor carboxyl terminus and correlates with receptor internalization. Mol. Endocrinol..

[bib38] Quitterer U., Fu X., Pohl A., Bayoumy K.M., Langer A., AbdAlla S. (2019). Beta-Arrestin1 prevents preeclampsia by downregulation of mechanosensitive AT1-B2 receptor Heteromers. Cell.

[bib39] Rakesh K., Yoo B., Kim I.M., Salazar N., Kim K.S., Rockman H.A. (2010). beta-Arrestin-biased agonism of the angiotensin receptor induced by mechanical stress. Sci. Signal..

[bib40] Ranjan R., Dwivedi H., Baidya M., Kumar M., Shukla A.K. (2017). Novel structural insights into GPCR-beta-arrestin interaction and signaling. Trends Cell Biol..

[bib41] Rozenfeld R., Gupta A., Gagnidze K., Lim M.P., Gomes I., Lee-Ramos D., Nieto N., Devi L.A. (2011). AT1R-CB(1)R heteromerization reveals a new mechanism for the pathogenic properties of angiotensin II. EMBO J..

[bib42] Santos R., Ursu O., Gaulton A., Bento A.P., Donadi R.S., Bologa C.G., Karlsson A., Al-Lazikani B., Hersey A., Oprea T.I. (2017). A comprehensive map of molecular drug targets. Nat. Rev. Drug Discov..

[bib43] Sato P.Y., Chuprun J.K., Schwartz M., Koch W.J. (2015). The evolving impact of g protein-coupled receptor kinases in cardiac health and disease. Physiol. Rev..

[bib44] Sauliere A., Bellot M., Paris H., Denis C., Finana F., Hansen J.T., Altie M.F., Seguelas M.H., Pathak A., Hansen J.L. (2012). Deciphering biased-agonism complexity reveals a new active AT1 receptor entity. Nat. Chem. Biol..

[bib45] Shenoy S.K., Lefkowitz R.J. (2011). beta-Arrestin-mediated receptor trafficking and signal transduction. Trends Pharmacol. Sci..

[bib46] Shin H.K., Kim Y.K., Kim K.Y., Lee J.H., Hong K.W. (2004). Remnant lipoprotein particles induce apoptosis in endothelial cells by NAD(P)H oxidase-mediated production of superoxide and cytokines via lectin-like oxidized low-density lipoprotein receptor-1 activation: prevention by cilostazol. Circulation.

[bib47] Shukla A.K., Kim J., Ahn S., Xiao K., Shenoy S.K., Liedtke W., Lefkowitz R.J. (2010). Arresting a transient receptor potential (TRP) channel: beta-arrestin 1 mediates ubiquitination and functional down-regulation of TRPV4. J. Biol. Chem..

[bib48] Siddiquee K., Hampton J., McAnally D., May L., Smith L. (2013). The apelin receptor inhibits the angiotensin II type 1 receptor via allosteric trans-inhibition. Br. J. Pharmacol..

[bib49] Smith J.S., Rajagopal S. (2016). The beta-arrestins: multifunctional regulators of G protein-coupled receptors. J. Biol. Chem..

[bib50] Steinberg D. (1997). Low density lipoprotein oxidation and its pathobiological significance. J. Biol. Chem..

[bib51] Steinberg D., Witztum J.L. (2010). Oxidized low-density lipoprotein and atherosclerosis. Arterioscler Thromb. Vasc. Biol..

[bib52] Sun S.W., Zu X.Y., Tuo Q.H., Chen L.X., Lei X.Y., Li K., Tang C.K., Liao D.F. (2010). Caveolae and caveolin-1 mediate endocytosis and transcytosis of oxidized low density lipoprotein in endothelial cells. Acta Pharmacol. Sin.

[bib53] Takezako T., Unal H., Karnik S.S., Node K. (2015). Structure-function basis of attenuated inverse agonism of angiotensin II type 1 receptor blockers for active-state angiotensin II type 1 receptor. Mol. Pharmacol..

[bib54] Tang W., Strachan R.T., Lefkowitz R.J., Rockman H.A. (2014). Allosteric modulation of beta-arrestin-biased angiotensin II type 1 receptor signaling by membrane stretch. J. Biol. Chem..

[bib55] Torisu K., Singh K.K., Torisu T., Lovren F., Liu J., Pan Y., Quan A., Ramadan A., Al-Omran M., Pankova N. (2016). Intact endothelial autophagy is required to maintain vascular lipid homeostasis. Aging Cell.

[bib56] Toshima S., Hasegawa A., Kurabayashi M., Itabe H., Takano T., Sugano J., Shimamura K., Kimura J., Michishita I., Suzuki T. (2000). Circulating oxidized low density lipoprotein levels. A biochemical risk marker for coronary heart disease. Arterioscler Thromb. Vasc. Biol..

[bib57] Turu G., Szidonya L., Gaborik Z., Buday L., Spat A., Clark A.J., Hunyady L. (2006). Differential beta-arrestin binding of AT1 and AT2 angiotensin receptors. FEBS Lett..

[bib58] Violin J.D., Lefkowitz R.J. (2007). Beta-arrestin-biased ligands at seven-transmembrane receptors. Trends Pharmacol. Sci..

[bib59] Vion A.C., Kheloufi M., Hammoutene A., Poisson J., Lasselin J., Devue C., Pic I., Dupont N., Busse J., Stark K. (2017). Autophagy is required for endothelial cell alignment and atheroprotection under physiological blood flow. Proc. Natl. Acad. Sci. U S A.

[bib60] Wang J., Hanada K., Gareri C., Rockman H.A. (2018). Mechanoactivation of the angiotensin II type 1 receptor induces beta-arrestin-biased signaling through Galphai coupling. J. Cell Biochem..

[bib61] Wassmann S., Czech T., van Eickels M., Fleming I., Bohm M., Nickenig G. (2004). Inhibition of diet-induced atherosclerosis and endothelial dysfunction in apolipoprotein E/angiotensin II type 1A receptor double-knockout mice. Circulation.

[bib62] Wingler L.M., Elgeti M., Hilger D., Latorraca N.R., Lerch M.T., Staus D.P., Dror R.O., Kobilka B.K., Hubbell W.L., Lefkowitz R.J. (2019). Angiotensin analogs with divergent bias stabilize distinct receptor conformations. Cell.

[bib63] Wootten D., Christopoulos A., Sexton P.M. (2013). Emerging paradigms in GPCR allostery: implications for drug discovery. Nat. Rev. Drug Discov..

[bib64] Yamamoto K., Kakino A., Takeshita H., Hayashi N., Li L., Nakano A., Hanasaki-Yamamoto H., Fujita Y., Imaizumi Y., Toyama-Yokoyama S. (2015). Oxidized LDL (oxLDL) activates the angiotensin II type 1 receptor by binding to the lectin-like oxLDL receptor. FASEB J..

[bib65] Zhang H., Unal H., Desnoyer R., Han G.W., Patel N., Katritch V., Karnik S.S., Cherezov V., Stevens R.C. (2015). Structural basis for ligand recognition and functional selectivity at angiotensin receptor. J. Biol. Chem..

[bib66] Zhang H., Unal H., Gati C., Han G.W., Liu W., Zatsepin N.A., James D., Wang D., Nelson G., Weierstall U. (2015). Structure of the Angiotensin receptor revealed by serial femtosecond crystallography. Cell.

[bib67] Zou Y., Akazawa H., Qin Y., Sano M., Takano H., Minamino T., Makita N., Iwanaga K., Zhu W., Kudoh S. (2004). Mechanical stress activates angiotensin II type 1 receptor without the involvement of angiotensin II. Nat. Cell Biol..

