# Supplementary material for: The endocytosis of oxidized LDL via the activation of the angiotensin II type 1 receptor
Source: iScience. 2021 Jan 21;24(2):102076. doi: 10.1016/j.isci.2021.102076 (PMC7890409; doi:10.1016/j.isci.2021.102076)
Supplement: Document S1. Transparent methods and Figures S1–S11 [file mmc1.pdf]

iScience, Volume *24*

Supplemental Information

The endocytosis of oxidized LDL via the activation of the angiotensin II type 1 receptor

Toshimasa Takahashi, Yibin Huang, Koichi Yamamoto, Go Hamano, Akemi Kakino, Fei Kang, Yuki Imaizumi, Hikari Takeshita, Yoichi Nozato, Satoko Nozato, Serina Yokoyama, Motonori Nagasawa, Tatsuo Kawai, Masao Takeda, Taku Fujimoto, Kazuhiro Hongyo, Futoshi Nakagami, Hiroshi Akasaka, Yoichi Takami, Yasushi Takeya, Ken Sugimoto, Herbert Y. Gaisano, Tatsuya Sawamura, and Hiromi Rakugi

Transparent Methods

Cell culture and materials

HUVECs and HAECs were cultured in EGM-2 (Clontech, USA). Cells less than five passages were used for the experiments. Transgenic CHO cells were maintained in F-12 Nutrient Mixture with Glutamax^{TM}-I (Thermo Fisher Scientific, USA), 10% fetal bovine serum (FBS), and appropriate selection reagents as described below. Transcription of the genes in transgenic CHO cells was induced by adding doxycycline into culture media for 24 h at a final concentration of 300 ng/ml. CHO-K1 cells were maintained in F-12 Nutrient Mixture with Glutamax^{TM}-I (Thermo Fisher Scientific, USA) and 10% FBS. barbadin was purchased from Toronto research chemicals (Canada). Losartan, telmisartan, and irbesartan were purchased from Cayman Chemical (USA).

Construction of plasmid vectors

For stable transformants, pTRE2hyg vector encoding mutated human AT1 with impaired ability to activate G protein (pTRE2hyg-HA-FLAG-hAT1mβ) and pTRE2hyg vector (Clontech, USA) encoding mutated hAT1 with impaired ability to activate β-arrestin (pTRE2hyg-HA-FLAG-hAT1mg) were created using site direct mutagenesis. Briefly, pTRE2hyg-HA-FLAG-hAT1mβ was created using a primeSTAR mutagenesis basal kit (Takara, Japan) to delete amino acids 221 and 222 from the pTRE2hyg vector encoding hAT1 tagged with signal peptide-HA-FLAG at the N-terminus (pTRE2hyg-HA-FLAG-hAT1) (Haendeler et al., 2000; Yamamoto et al., 2015). pTRE2hyg-HA-FLAG-hAT1mg was created using a KOD-plus mutagenesis kit (Toyobo, Japan) to substitute amino acids at the carboxyl terminus (Thr (332), Ser (335), Thr (336), Ser (338)) into alanine from pTRE2hyg-HA-FLAG-hAT1 (Qian et al., 2001). For the BRET assay, human β-arrestin

2 was subcloned into mVenus N1 (Plasmid #27793, Addgene). Expression vectors for LOX-1 and Dectin-1 were created as shown in a previous study (Yamamoto et al., 2015). For real-time imaging, LOX-1 tagged with V5-6×His at the C-terminus (V5-LOX-1) was subcloned into pmScarlet_C1 (Plasmid #85042, Addgene) (mScarlet-LOX-1). HA-FLAG-hAT1, HA-FLAG-hAT1mβ, and HA-FLAG-hAT1mg were subcloned into pcDNA3-EGFP (Plasmid #85042, Addgene) (AT1-GFP, AT1mg-GFP, and AT1mβ-GFP). Plasmid encoding dominant-negative β-arrestin was created by subcloning the clathrin-binding domain of β-arrestin (β-arrestin (319–418)) into the pTRE2hyg vector (DN-βarrestin) (Krupnick et al., 1997). Negative control vector of DN-β-arrestin was created using primeSTAR mutagenesis basal kit to delete the clathrin binding box domain (LIELD) from DN-β-arrestin (Kang et al., 2009).

Stable transformants

CHO-K1 cells expressing tetracycline-inducible human LOX-1 tagged with V5-6×His at C-terminus (CHO-LOX-1), cells expressing human HA-FLAG-hAT1 (CHO-AT1), and cells expressing both human LOX-1 and AT1 (CHO-LOX-1-AT1) were maintained as previously described (Fujita et al., 2009; Yamamoto et al., 2015). To establish cells expressing both LOX-1 and mutated AT1, pTRE2hyg-HA-FLAG-hAT1mβ or pTRE2hyg-HA-FLAG-hAT1mg were co-transfected with pSV2bsr vector (Funakoshi, Japan) into CHO-LOX-1 using Lipofectamin2000 transfection reagent (Thermo Fisher Scientific, USA). The stable transformants were selected with 400 μg/ml of hygromycin B (Wako, Japan) and 10 μg/ml of blasticidin S (Funakoshi, Japan). The resistant clones expressing LOX-1 and mutated AT1 in response to doxycycline (Calbiochem, USA) were selected for use in experiments (CHO-LOX-1-AT1mg and CHO-LOX-1-AT1mβ).

Immunofluorescence staining

Tagged LOX-1 and AT1 (or mutated AT1) in genetically engineered CHO cells were detected using mouse anti-V5 (Nacalai, Japan) and rat anti-FLAG (Novus Biologicals, USA) antibodies in combination with rabbit Alexa488-conjugated anti-rat IgG and goat Alexa594-conjugated anti-mouse IgG (Thermo Fisher Scientific, USA), respectively, as reported previously (Yamamoto et al., 2015). Nuclei were counterstained with DAPI (Sigma, USA). Images were acquired with a fluorescence microscope (BZ-X700, Keyence, Japan).

Cell-based ELISA

Cells were seeded at 150,000 cells per well onto 96-well transparent cell culture plates and incubated overnight at 37°C. The following day, cultures were transferred to serum-free conditions and cells were further incubated for 24 h. Thereafter, cells were fixed by 4% paraformaldehyde without permeabilization, incubated with mouse anti-V5 or rat anti-FLAG antibodies, then incubated with HRP-conjugated mouse or rat secondary antibodies, respectively. TMB reagents (SeraCare Life Sciences, USA) were then added to each well and the colorimetric reaction was stopped with stop solution (SeraCare Life

Sciences, USA). OD 450 values were measured using Multiskan Go (Thermo Fisher Scientific, USA). Each measurement value was adjusted by subtracting the value of negative control with secondary antibodies in the absence of first antibodies.

***In situ* PLA**

In situ PLA was used to detect the proximity of LOX-1 with AT1 or mutated AT1 using Duolink, from Olink Bioscience (Uppsala, Sweden), according to our previous study (Yamamoto et al., 2015). Images were acquired using a fluorescence microscope (BZ-X700, Keyence, Japan). Quantitative fluorescence cell image analysis was performed using the BZ-X analyzer system (Keyence, Japan).

Preparation of oxLDL and fluorescence-labelled oxLDL

Human plasma LDL (1.019-1.063 g/ml) isolated by sequential ultracentrifugation was oxidized using 20 μM $CuSO_4$ in PBS at 37°C for 24 h. Oxidation was terminated by adding excess EDTA. Oxidation of LDL was analyzed using agarose gel electrophoresis for migration versus LDL (Yamamoto et al., 2015). Labeling of oxLDL with 1,1-dioctadecyl-3,3,3,3-tetramethylindocarbocyanine perchlorate (DiI, Thermo Fisher

Scientific, USA) was performed as described previously (Yamamoto et al., 2015).

Quantification of cellular cAMP content

Gi-dependent inhibition of adenylyl cyclase activity was assessed by inhibition of Forskolin-dependent cAMP production using a cAMP dynamic 2 kit (Cisbio, France). Cells were seeded at 80,000 cells per well onto 96-well transparent cell culture plates and incubated overnight at 37°C. The following day, cultures were transferred to serum-free conditions and cells were further incubated for 24 h. Thereafter, cells were treated for 1 h with DMEM without phenol, 1 mM IBMX, and 1 μM Forskolin, including vehicle, oxLDL, and AII at the indicated concentrations at 37°C, 5% CO_2. Triton X was then added to a final concentration of 1% and cell lysates were prepared after shaking the plates for 30 min. Finally, cell lysates were transferred to 384-well white plates and cAMP levels were measured by incubation of cell lysates with FRET reagents (the cryptate-labeled anti-cAMP antibody and the d2-labeled cAMP analogue) for 1 h at 37°C. The emission signals were measured at 590 and 665 nm after excitation at 340 nm, using the ARTEMIS plate reader (Furuno Electric Co. Ltd, Japan). The FRET ratio: F = (fluorescence 665 nm/fluorescence 590 nm) $\times 10^4$ was transformed into cAMP concentration by calculation using the four-parameter logistic curve of standard samples.

Measurement values were normalized to that of vehicle treatment.

Quantification of cellular IP1 accumulation

Gq-dependent activation of phospholipase C was quantified by measurement of IP1 using the IP-One assay kit (Cisbio, France). Cells were seeded at 80,000 cells per well onto 96-well transparent cell culture plates and incubated overnight at 37°C. The following day, cultures were transferred to serum-free conditions and further incubated for 24 h. Thereafter, cells were treated for 1 h with IP1 stimulation buffer mixed with the same amount of DMEM without phenol, including vehicle, oxLDL, and AII at an indicated concentration at 37°C, 5% CO_2. Triton X was then added to a final concentration of 1%, and cell lysates were prepared after shaking the plates for 30 min. Finally, cell lysates were transferred to a 384-well white plate and IP1 levels were measured by incubation of cell lysates with FRET reagents (the cryptate-labeled anti-IP1 antibody and the d2-labeled IP1 analogue) for 1 h at 37°C. The emission signals were measured at 590 and 665 nm after excitation at 340 nm, using the ARTEMIS plate reader (Furuno Electric Co. Ltd., Japan). The FRET ratio: F= (fluorescence 665 nm/fluorescence 590 nm) $\times 10^4$ was transformed into IP1 concentration by calculation using the four-parameter logistic curve of standard samples. Measurement values were normalized to that of vehicle treatment.

Real-time imaging of dynamics in LOX-1 and AT1 on cellular membranes

Twenty-four hours before imaging experiments, CHO cells were transfected with LOX-1-mScarlet and mock-GFP AT1-GFP or AT1 mutants-GFP by electroporation and seeded in a 35 mm glass base dish (Iwaki, Japan) pre-coated with 1000X diluted 10 mg/ml Poly-L-Lysine (ScienCell, USA) 1 h before seeding. The growth medium was replaced with imaging buffer (pH 7.4) containing 125 mM NaCl, 5 mM KCl, 1.2 mM $MgCl_2$, 1.3 mM $CaCl_2$, 25 mM HEPES, and 3 mM D-glucose with pH adjusted to 7.4 with NaOH. Dynamic images of the cells were obtained at 25°C using SpinSR10 inverted spinning disk-type confocal super-resolution microscope (Olympus, Japan) equipped with a 100x NA1.49 objective lens (UAPON100XOTIRF, Olympus, Japan) and an ORCA-Flash 4.0 V2 scientific CMOS camera (Hamamatsu Photonics KK, Japan) at 5 s intervals. The imaging experiment was performed with CellSens Dimension 1.11 software using 3D deconvolution algorithm (Olympus, Japan).

Quantification of change in LOX-1 localization on cellular membrane

A count of puncta was performed using separated images visualizing LOX-1-scarlet just before (0 min) and 3 min after ligand application. Puncta was manually counted by a

blinded observer, and number of puncta at 0 and 3 min was determined (N0 and N3, respectively). Change in puncta was calculated as (N0-N3)/N0 (Fig 4a).

Detection of membrane-bound oxLDL

Genetically engineered CHO cells were treated for 30 min with Dil-labeled oxLDL at a final concentration of 2 μg/ml on ice, as described previously (Yamamoto et al., 2015). The cells were then washed twice and fixed with neutral buffered formalin. Nuclei were stained with DAPI (1 μg/ml). Images were acquired using a fluorescence microscope (BZ-X700, Keyence, Japan). Quantitative fluorescence cell image analysis was performed using the BZ-X analyzer system (Keyence, Japan).

Visualization of co-localization of oxLDL with endocytic organelles and lysosome

CHO-LOX-1-AT1 cells and HUVECs were seeded in a 35 mm four well glass base dish (Iwaki, Japan) pre-coated with 1000X diluted 10 mg/ml Poly-L-Lysine (ScienCell, USA) 1 h before seeding. The following day, cells were treated with CellLight® for early and late endosomes and lysosomes, according to the instructions (Thermo Fisher Scientific, USA). After 24 h, cells were treated for 30 min with Dil-labeled oxLDL at a concentration of 2 μg/ml. The cells were then washed twice with cultured media and fixed with neutral

buffered formalin immediately or after 5 h incubation in a CO_2 incubator. Images were obtained using a SpinSR10 inverted spinning disk-type confocal super-resolution microscope (Olympus, Japan) equipped with a 100x NA1.49 objective lens (UAPON100XOTIRF, Olympus, Japan) and an ORCA-Flash 4.0 V2 scientific CMOS camera (Hamamatsu Photonics KK, Japan). The imaging experiment was performed with the CellSens Dimension 1.11 software using 3D deconvolution algorithm (Olympus, Japan).

Detection of colocalization of endosomes or lysosomes with Dil-oxLDL

Before determining sub-pixel localization of lysosomes and endosomes, obtained raw images were processed by despeckle and à trous wavelet transform algorithm to reduce noise and remove the background with Fiji. Using custom MATLAB software, the weighted centroid sub-pixel localizations were detected automatically. We confirmed these results point-by-point to remove any possible artifacts then quantified the colocalizations. Based on the resolution limit and configuration of our microscope, we defined a 300 nm radius as the cut-off of subpixel colocalization.

Detection of intracellular oxLDL content

Genetically engineered CHO cells seeded in 96-well plates were treated for 30 min with Dil-labeled oxLDL at a concentration of 2 µg/ml unless otherwise indicated in a CO_2 incubator at 37°C. HUVECs and HAECs in 96-well plates were treated for the indicated time duration (10min, 30min, or 6 h) with Dil-labeled oxLDL at a concentration of 2 µg/ml in a CO_2 incubator. The cells were then washed twice with cultured media and further incubated overnight to wash out the membrane-bound oxLDL. The cells were then washed twice and fixed with neutral buffered formalin. Nuclei were stained with DAPI (1 µg/ml). Images were acquired using a fluorescence microscope (BZ-X700, Keyence, Japan). Quantitative fluorescence cell image analysis was performed using the BZ-X analyzer system (Keyence, Japan).

Transfection of CHO cells with dominant negative β-arrestin

Genetically engineered CHO cells were transfected with DN-β-arrestin or negative control vector using Lipofectamin LTX & PLUS reagent (Thermo Fisher Scientific, USA), according to the manufacturer’s instructions. Treatment with Dil-oxLDL was performed 24 h after transfection.

Luciferase reporter assay

Cells were seeded in a 96-well plate and were cotransfected with 100 ng of a plasmid containing Firefly luciferase driven by the NF-κB binding site (Promega, USA), and 10 ng of pRL-CMV Renilla luciferase control reporter vector (Promega Corp., Madison, WI, USA) by using Lipofectamine LTX with PLUS reagent kit (Thermo Fisher Scientific). After being cultured for 6 hours, the cells were starved in 0.1 % FBS supplemented with 300 ng/mL doxycycline to induce LOX-1 expression for 24 hours. Then, oxLDL was added to cells and incubated for 24 hours. Cells were washed once with PBS and lysed by incubation with 150 µL Passive Lysis Buffer from the Dual-Luciferase Reporter Assay Kit (Promega) for 15 minutes at room temperature with mixing. Lysates (10 µL) were loaded onto a 96-well white plate, and firefly and Renilla luciferase activities were determined. Luminescence was measured by using a Spark® microplate reader (TECAN, Switzerland)

Transfection of human endothelial cells with siRNA

HUVECs and HAVSMCs were plated to be 50% confluent on the day of transfection. Silencer® select siRNA for LOX-1 and/or AT1 (Thermo Fisher Scientific, USA) was transfected into the cells in media without serum and antibiotics using lipofectamine

RNAiMAX (Thermo Fisher Scientific, USA), according to the manufacturer's instructions. Treatment with Dil-oxLDL was performed 24 h after transfection.

Quantitative real-time PCR

Total RNA was extracted using an RNeasy Mini Kit (Thermo Fisher Scientific, USA) with DNase I treatment, and an equivalent amount of RNA was transcribed to cDNA by the RevetraAce qPCR RT kit (FSQ-101, TOYOBO, Japan). Quantitative real-time PCR was performed and analyzed on a model 7900 sequence detector (Thermo Fisher Scientific, USA) using TaqMan gene-expression assays for *LOX-1* (Hs01552593_m1) and *AT1* (5'-ACGTGTCTCAGCATTGATCGAT-3' and 5'-GTCGAAGGCGGGACTTCA-3' for primers, and 5'-CCTGGCTATTGTTCACC-3' for probe), or the SYBR green qPCR system (Thermo Fisher Scientific, USA) with specific primer pairs for *SCARB1* (5'-CTGTGGGTGAGATCATGTGG-3' and 5'-GCCAGAAGTCAACCTTGCTC-3'), *ARRB1* (5'-GGAGAACCCATCAGCGTCAA-3' and 5'-GGGCACTTGTACTGAGCTGT-3'), *ARRB2* (5'-CAACTCCACCAAGACCGTCAAGA-3' and 5'-TTCGAGTTGAGCCACAGGACACTT-3'), *GRK2* (5'-ATGCATGGCTACATGTCCA-3' and 5'-ATCTCCTCCATGGTCAGCAG-3'), *GRK3* (5'-

AGCTGTAGAACACGTACAAAGTC-3' and 5'-ATGTCACCTCGAAGGCTTTCA-3'), *GRK5* (5'-ACCTGAGGGGAGAACCATTC-3' and 5'-TGGACTCCCCTTTCCTCTTT-3'), *GRK6* (5'-TAGCGAACACGGTGCTACTC-3' and 5'-GCTGATGTGAGGGAACTGGA-3'), *CLTC* (5'-GCCAGATGTCGTCCTGGAAA-3' and 5'-AGCTGGGGCTGACCATAAAC-3'), and *CAV1* (5'-CCAAGGAGATCGACCTGGTCAA-3' and 5'-GCCGTCAAAACTGTGTGTCCCT-3') The expression level of each gene was determined by the standard curve method and normalized using *GAPDH* mRNA (5'-GCCATCAATGACCCCTTCATT-3' and 5'-TCTCGCTCCTGGAAGATGG-3') as an internal control.

Detection of phosphorylation of ERK1/2

Cells treated with oxLDL or vehicle were kept in an incubator at 37°C for 10 min. Subsequently, cells were washed twice with PBS, and lysed using M-PER Mammalian Protein Extraction Reagent (Thermo Scientific, Waltham, MA, USA) with protease inhibitor and phosphatase inhibitor followed by Western blotting analysis as described below.

Western blotting analysis

Proteins were separated by SDS-PAGE and electrophoretically transferred to polyvinylidene fluoride membranes for Western blot analysis. The membranes were

blocked with 5% nonfat dried milk and incubated with primary antibodies overnight at 4ºC. The primary antibodies used in this study were anti–phospho-ERK1/2 (Thr202/Tyr204) antibody, anti–total-ERK1/2 antibody (Cell Signaling Technology, Danvers, MA, USA). Bands were visualized using Chemi-Lumi One Super (Nacalai Tesque). Densitometoric analysis was performed with chemiluminescence detection system (LAS-4000 mini, GE Healthcare, Pittsburgh, PA, USA)

Bioluminescence resonance energy transfer assay to monitor AT1-β-arrestin interaction

CHO-K1 cells were seeded onto a 35 mm dish at a density of $3x10^5$ cells. The following day, cells were transfected with AT1-rluc, β-arrestin2-mVenus, and non-fluorescence-labelled LOX-1, or Dectin-1 of 0.9, 0.3, and 1.8 µg, respectively, using Lipofectamin LTX & PLUS reagent (Thermo Fisher Scientific, USA), according to the manufacturer's instructions. Cells were also transfected with AT1-rluc and non-fluorescence-labelled Dectin-1 of 0.9 and 1.8 µg without β-arrestin2-mVenus. Cultures were transferred to serum-free conditions after 24 h of transfection and further incubated for 24 h. Thereafter, cells were prepared in white clear-bottom 96-well plates at a density of 100,000 cells per well. Coelenterazine was added to each well at a final concentration of 5 µM and assays

were carried out immediately on a Spark® microplate reader (TECAN, Switzerland), and the BRET ratio (emission mVenus/emission Rluc) was calculated. After 3 min of reading of the baseline (the final baseline reading is presented at 0), cells were exposed to vehicle, oxLDL (100 μg/ml), SII (10–5 M), or AII (10–5 M) for 10 min. The relative change in intramolecular BRET ratio was calculated by subtracting the average BRET ratio measured for cells stimulated with vehicle.

Statistical analyses

All data are presented as the mean ± SEM. Significant differences between two treatments or among multiple treatments were determined by Student's t-test or one-way ANOVA with Bonferroni testing, respectively.

References

Fujita, Y., Kakino, A., Nishimichi, N., Yamaguchi, S., Sato, Y., Machida, S., Cominacini, L., Delneste, Y., Matsuda, H., and Sawamura, T. (2009). Oxidized LDL receptor LOX-1 binds to C-reactive protein and mediates its vascular effects. Clin Chem *55*, 285-294.

Haendeler, J., Ishida, M., Hunyady, L., and Berk, B.C. (2000). The third cytoplasmic loop of the angiotensin II type 1 receptor exerts differential effects on extracellular signal-regulated kinase (ERK1/ERK2) and apoptosis via Ras- and Rap1-dependent pathways. Circ

Res *86*, 729-736.

Kang, D.S., Kern, R.C., Puthenveedu, M.A., von Zastrow, M., Williams, J.C., and Benovic, J.L. (2009). Structure of an arrestin2-clathrin complex reveals a novel clathrin binding domain that modulates receptor trafficking. J Biol Chem *284*, 29860-29872.

Krupnick, J.G., Santini, F., Gagnon, A.W., Keen, J.H., and Benovic, J.L. (1997). Modulation of the arrestin-clathrin interaction in cells. Characterization of beta-arrestin dominant-negative mutants. J Biol Chem *272*, 32507-32512.

Qian, H., Pipolo, L., and Thomas, W.G. (2001). Association of beta-Arrestin 1 with the type 1A angiotensin II receptor involves phosphorylation of the receptor carboxyl terminus and correlates with receptor internalization. Mol Endocrinol *15*, 1706-1719.

Yamamoto, K., Kakino, A., Takeshita, H., Hayashi, N., Li, L., Nakano, A., Hanasaki-Yamamoto, H., Fujita, Y., Imaizumi, Y., Toyama-Yokoyama, S.*, et al.* (2015). Oxidized LDL (oxLDL) activates the angiotensin II type 1 receptor by binding to the lectin-like oxLDL receptor. FASEB J *29*, 3342-3356.

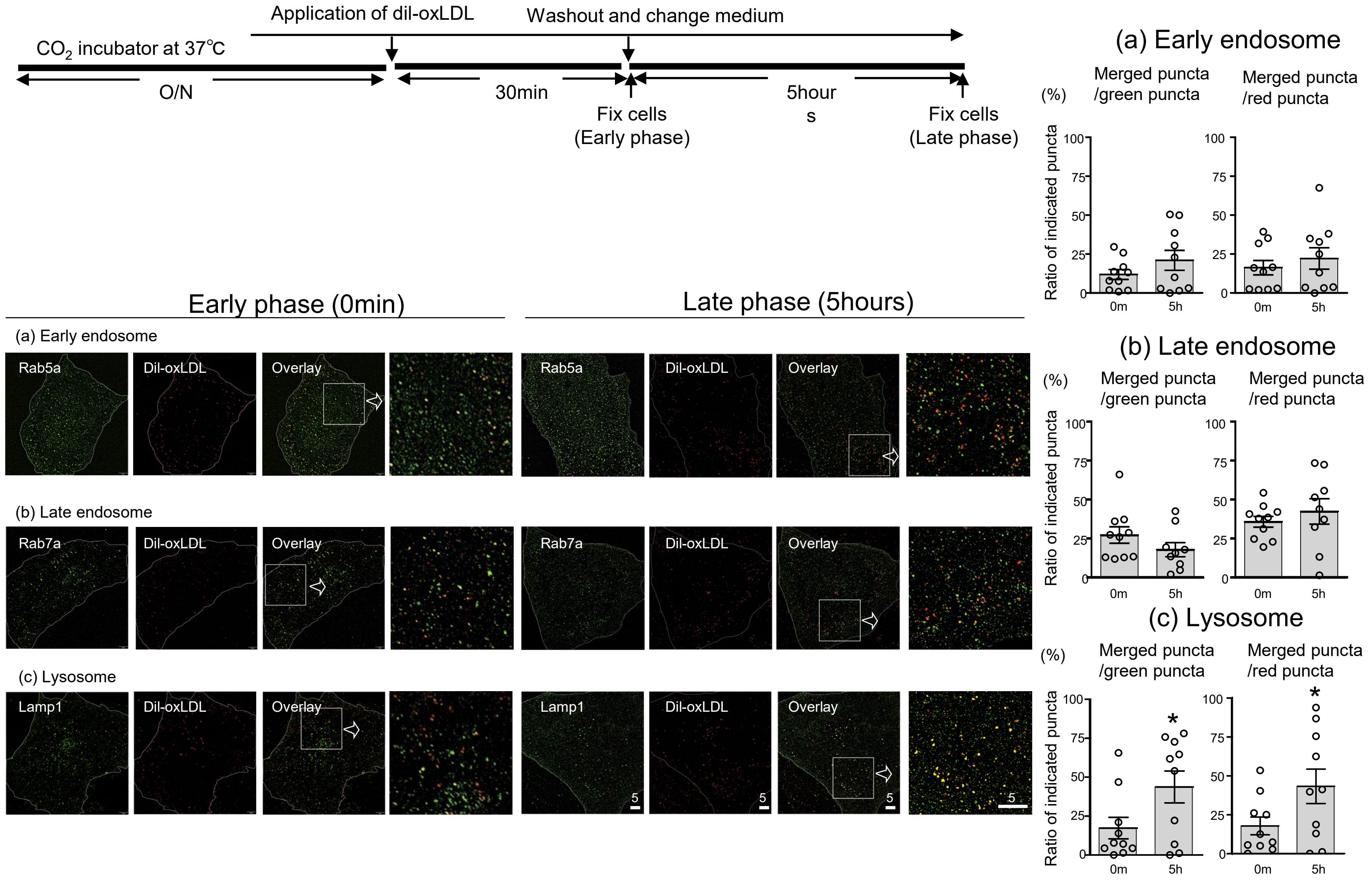

Figure S1: Endocytic traffic of oxLDL with early endosomes, late endosomes and lysosome in CHO. Related to Figure 5.

Visualization of co-localization of oxLDL with (a) early and (b) late endosomes, and (c) lysosomes using a confocal super-resolution microscope. Endosomes and lysosomes were visualized by transduction with a viral vector encoding GFP-fused indicated proteins in CHO-LOX-1-AT1 (CellLight™, Thermo Fisher Scientific, USA). After 30 min of treatment with 2 μg/ml Dil-labeled oxLDL, cells were washed and fixed immediately (early phase, 0 min) or after 5 h of additional incubation without oxLDL (late phase, 5 h). Approximate cell boundaries are marked with dotted lines. Yellow (merged) puncta in overlaid images indicate co-localization of oxLDL with each organelle.

(left lower panels) Representative images, scale bar, μm. (right panels) Quantification of proportion of merged puncta relative to total green (early, late endosomes or lysosomes) or red puncta (oxLDL) (n = 9–10, each group), analyzed as described in the methods. 0 m, early phase (0 min); 5 h, late phase (5 h);.Data are represented as mean +/- SEM. The differences were determined by Student's t-test. $^{*}p < 0.05$ vs. the others

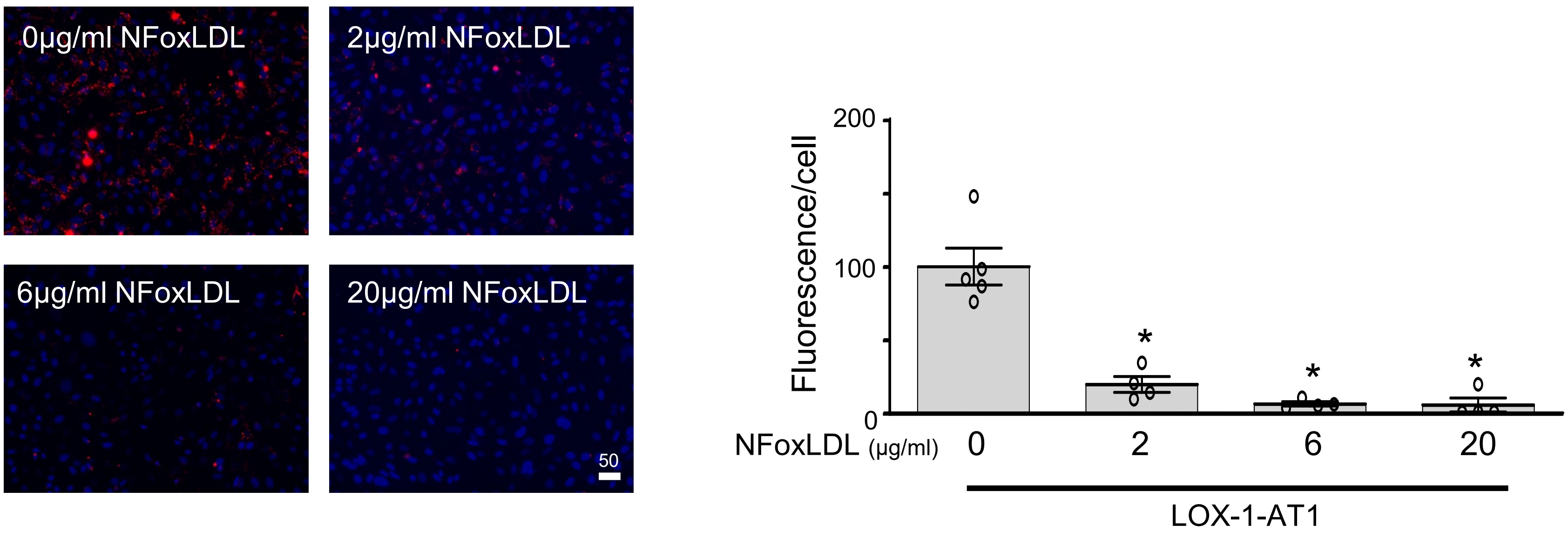

Figure S2: Cellular accumulation of Dil-oxLDL was blocked by non fluorescent oxLDL in CHO. Related to Figure 5.

Intracellular uptake of Dil-labeled oxLDL in CHO-LOX-1-AT1 with or without simultaneous treatment with non-fluorescent oxLDL (n = 4, each). Scale bar, μm

The graph indicates the fluorescence/number of nuclei, and the average value of the group at the far left was normalized to 100%. Data are represented as mean +/- SEM. The differences were determined by one-way ANOVA with Bonferroni correction. $^{*}p < 0.01$ vs. treatment without non-fluorescent oxLDL

NF-oxLDL, non-fluorescent oxLDL

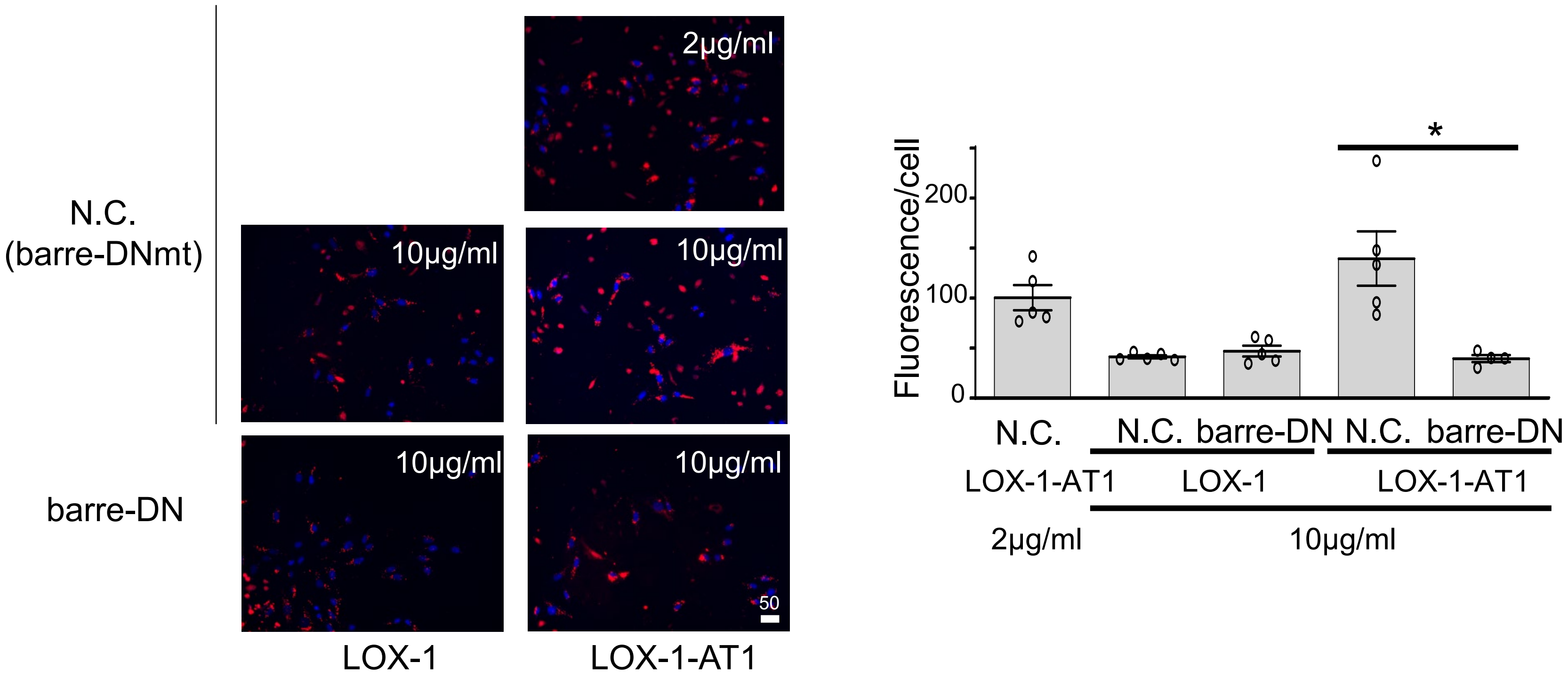

Figure S3: AT1-independent uptake of a higher concentration of oxLDL via LOX-1 was β-arrestin independent in CHO. Related to Figure 5.

Intracellular uptake of Dil-labeled oxLDL at the indicated concentrations in CHO-LOX-1 or CHO-LOX-1-AT1 transfected with dominant negative vector of β-arrestin (barre-DN) or negative control vector (N.C.) (n = 5, each). Scale bar, μm

The graph indicates the fluorescence/number of nuclei, and the average value of the group at the far left was normalized to 100%. Data are represented as mean +/- SEM. The differences were determined by one-way ANOVA with Bonferroni correction. $^{*}p < 0.01$

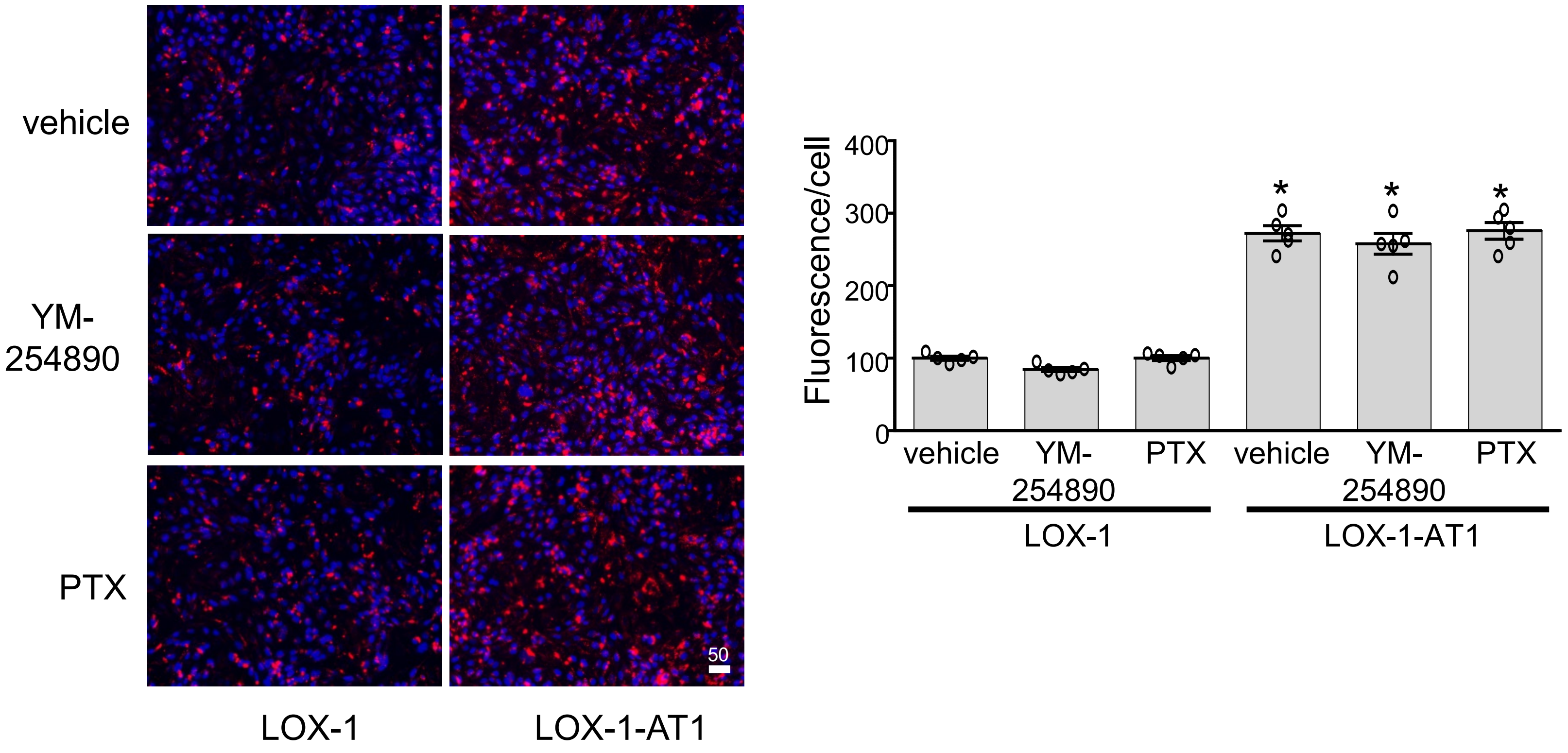

Figure S4: Cellular accumulation of oxLDL was independent of Gαi and Gαq-dependent pathways in CHO. Related to Figure 5.

Intracellular uptake of Dil-labeled oxLDL at the indicated concentrations in CHO-LOX-1 or CHO-LOX-1-AT1 pretreated with vehicle, Gq inhibitor, YM254890, or Gi inhibitor, pertussis toxin (PTX) (n = 5, each). Scale bar, μm YM-254890 and PTX were pretreated at 1 μM and 25 ng/ml for 30 min and 12 h before stimulation, respectively. The graph indicates the fluorescence/number of nuclei, and the average value of the group at the far left was normalized to 100%. Data are represented as mean +/- SEM. The differences were determined by one-way ANOVA with Bonferroni correction. $^{*}p < 0.01$ vs. treatment-matched wells in CHO-LOX-1

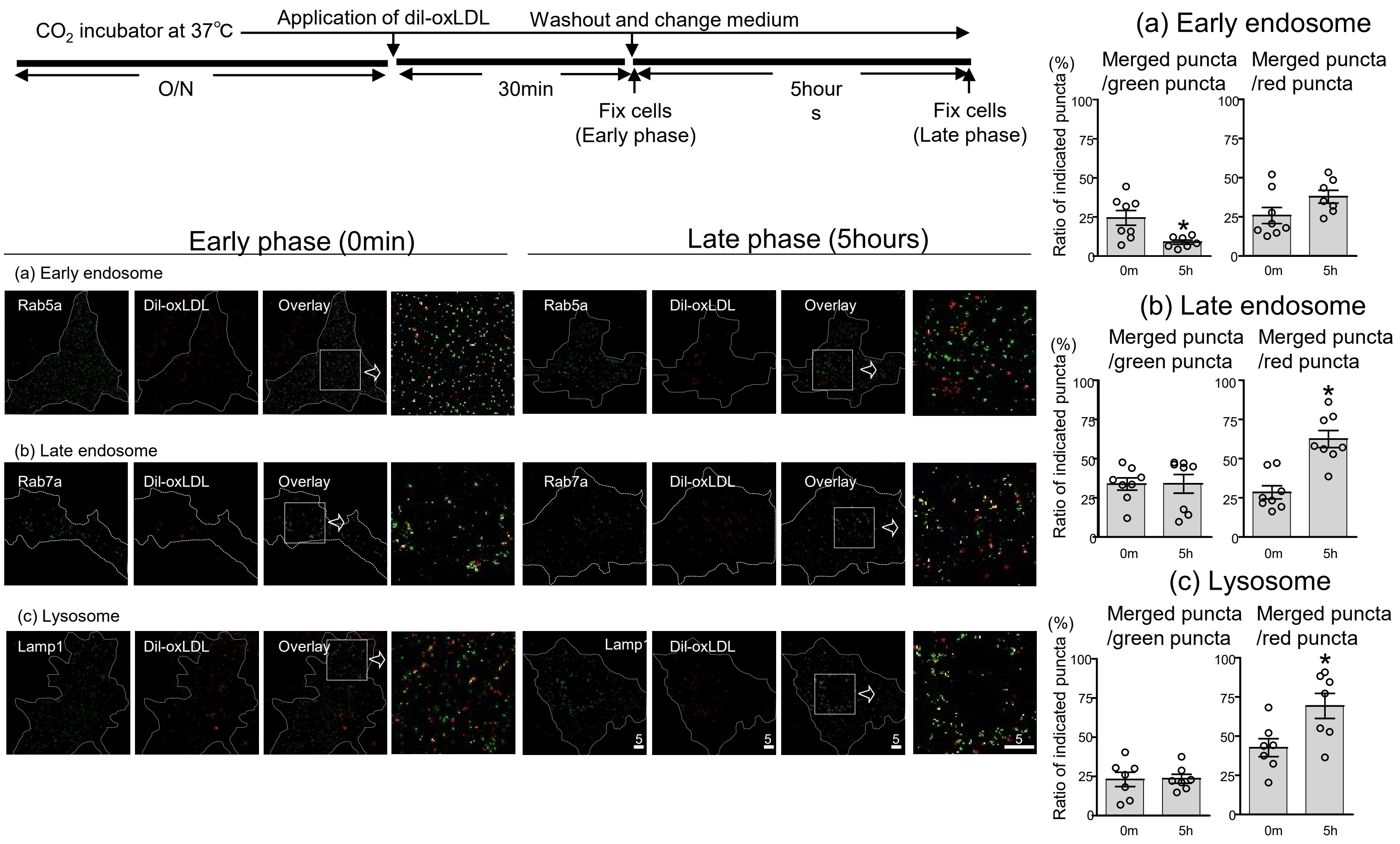

Figure S5: Endocytic traffic of oxLDL with early endosomes, late endosomes and lysosome in HUVECs. Related to Figure 6.

Visualization of co-localization of oxLDL with (a) early and (b) late endosomes, and (c) lysosomes using a confocal super-resolution microscope. Endosomes and lysosomes were visualized after transduction of a viral vector encoding GFP-fused indicated proteins in human umbilical vein endothelial cells (HUVECs) (CellLight™, Thermo Fisher Scientific, USA). After 30 min of treatment with 2 µg/ml Dil-labeled oxLDL, cells were washed and fixed immediately (early phase, 0 min) or after 5 h of additional incubation without oxLDL (late phase, 5 h). Approximate cell boundaries are marked with dotted lines. Yellow (merged) puncta in overlaid images indicate co-localization of oxLDL with each organelle.

(left lower panels) Representative images, scale bar, µm. (right panels) Quantification of proportion of merged puncta relative to total green (early, late endosomes or lysosomes) or red puncta (oxLDL) (n = 9–10, each group), analyzed as described in the methods. 0 m, early phase (0 min); 5 h, late phase (5 h);.Data are represented as mean +/- SEM. The differences were determined by Student's t-test. $^{*}p < 0.05$ vs. the others

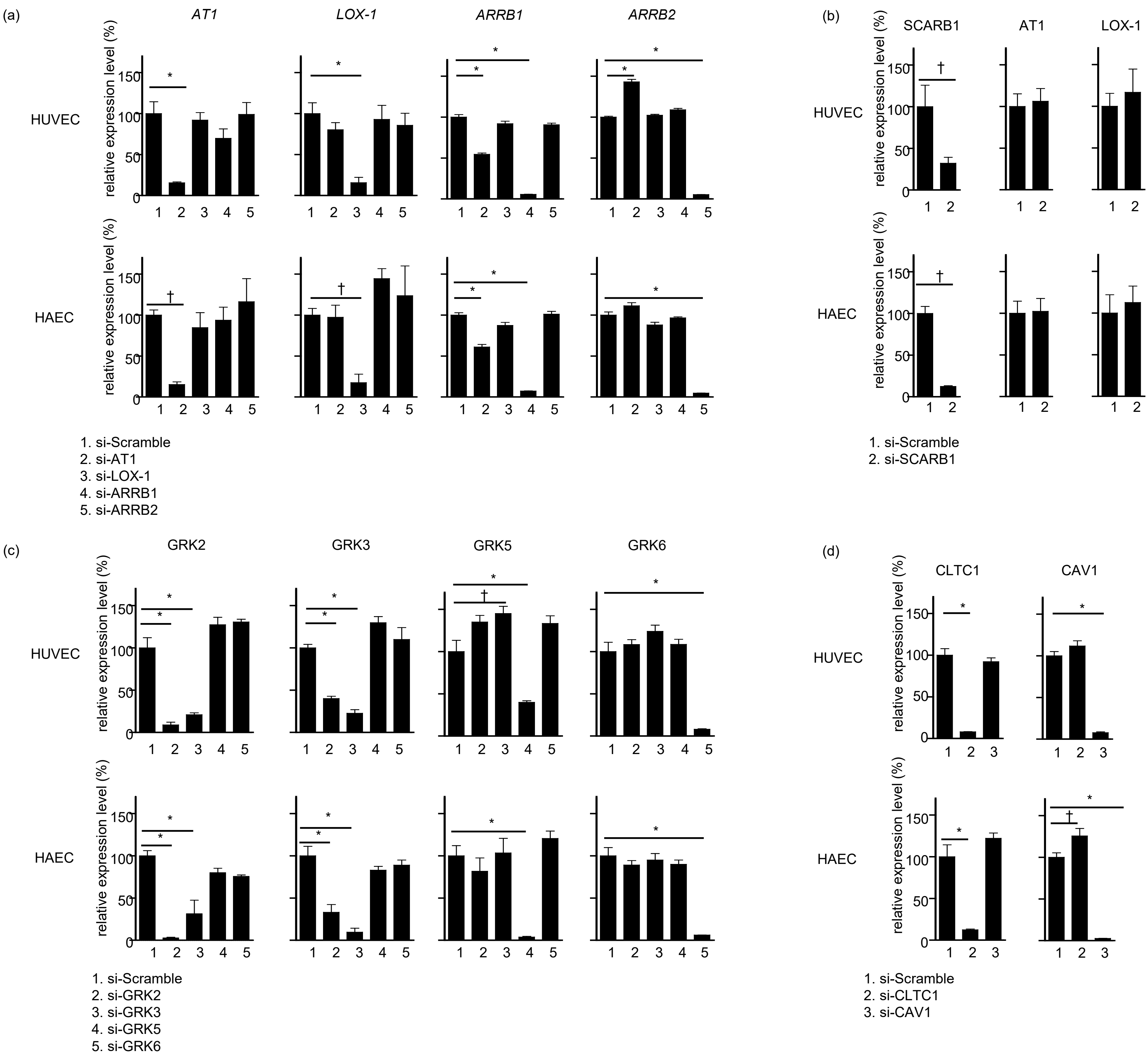

Figure S6: The siRNA-mediated knockdown efficiency of indicated genes in HUVECs and HAECs. Related to Figure 6.

(a–d) Confirmation of the efficiency of siRNA-mediated knockdown of indicated genes by quantitative real-time PCR in human umbilical vein endothelial cells (HUVECs) and human aortic endothelial cells (HAECs) (n = 4–5, each).

(a) siRNA against *AT1* decreased *ARRB1* expression in HUVECs and HAECs, and increased *ARRB2* expression in HUVECs, suggesting an undetermined interaction at the transcriptional level. (c) siRNA against *GRK2* or *GRK3* decreased GRK3 or GRK2 levels in HUVECs and HAECs, respectively, suggesting an interaction at the transcriptional level. The expression level of each gene was normalized using *GAPDH* mRNA as an internal control. Data are represented as mean +/- SEM. Differences with respect to the relative gene expression of cells treated with scramble siRNA (si-scramble) were determined by one-way ANOVA with Bonferroni correction. $^{*}p < 0.01$, $†p < 0.05$

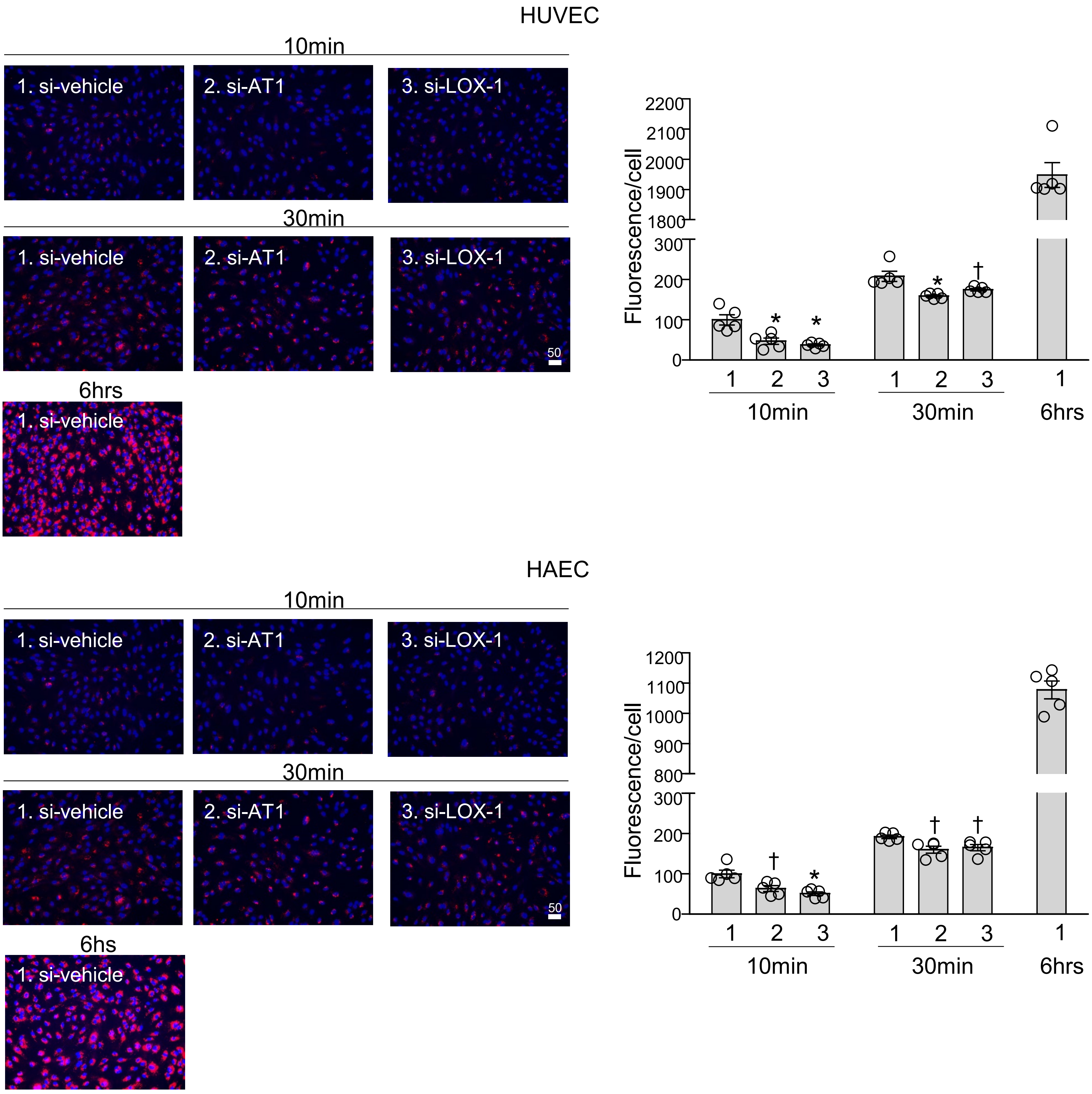

Figure S7: The inhibitory effect of siRNA against AT1 or LOX-1 in HUVECs and HAECs. Related to Figure 6.

Intracellular uptake of 2 μg/ml Dil-labeled oxLDL treated for three different durations to human umbilical vein endothelial cells (HUVECs) and human aortic endothelial cells (HAECs) with siRNA-mediated knockdown of the indicated genes (n = 5, each). Scale bar, μm

The graph indicates the fluorescence/number of nuclei, and the average value of the group at the far left was normalized to 100%. Data are represented as mean +/- SEM. The differences were determined by one-way ANOVA with Bonferroni correction $^{*}p < 0.01$ vs. control in each time course, †$p < 0.05$ vs. control in each time course

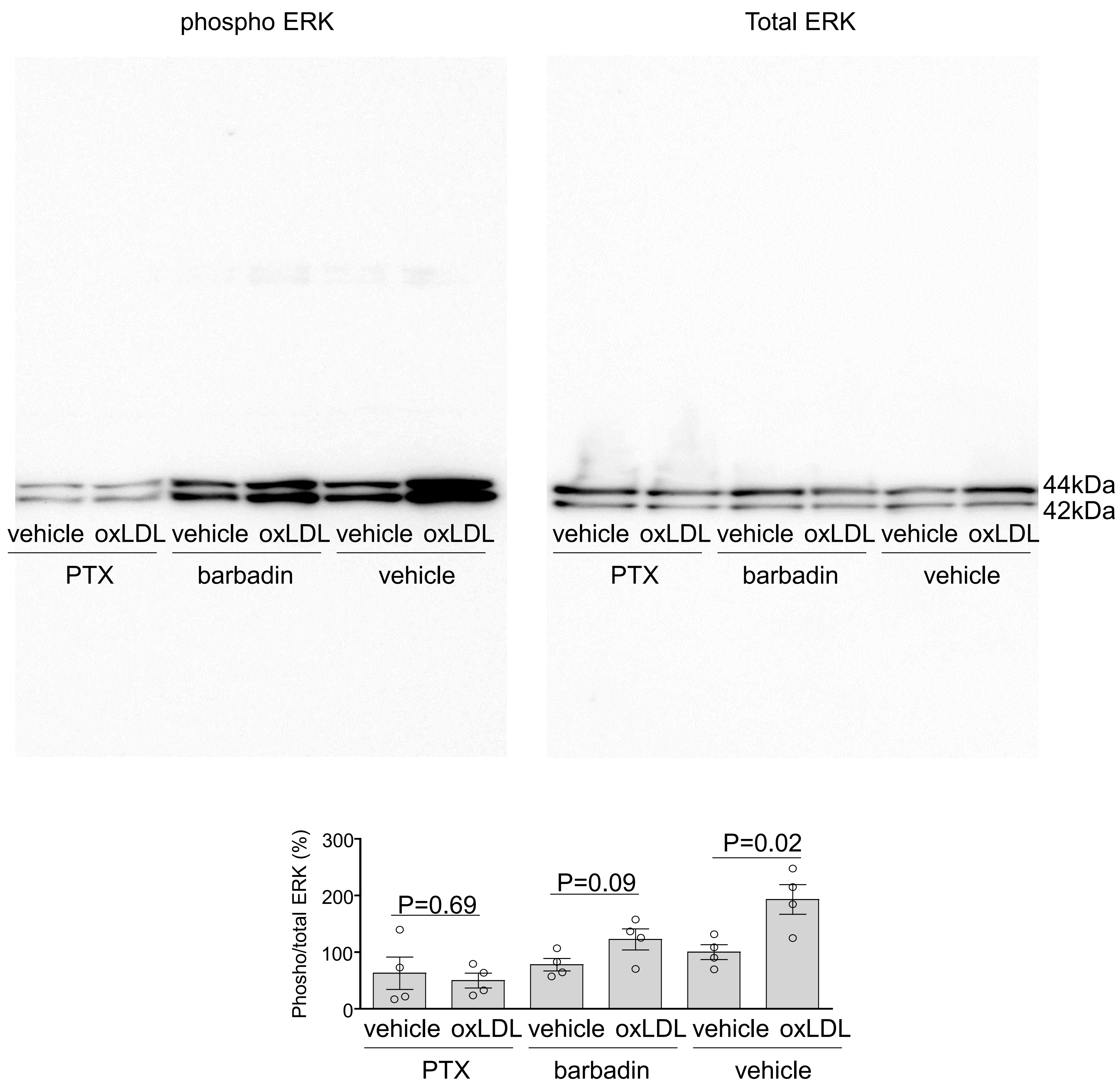

Figure S8: ERK1/2 activation induced by oxLDL was inhibited either by a Gi-specific inhibitor or a β-arrestin inhibitor in HUVECs. Related to Figure 6.

Immunoblotting for detecting phosphorylation of extracellular signal-regulated kinase 1/2 in human umbilical vein endothelial cells (HUVECs). Cells were stimulated with vehicle or 20μg/ml oxLDL for 10 min after a 6-h pre-treatment with vehicle, 25 ng/ml PTX, a Gi inhibitor, or 10 μM barbadin, a β-arrestin inhibitor. (Upper panel) Representative immunoblots of phosphorylated ERK1/2 and total ERK1/2. (Lower panel) Quantification of ERK 1/2 activation by densitometric analysis of phosphorylated/total ERK 1/2. The average activation after vehicle treatment following vehicle pretreatment was set at 100%. Data are represented as mean +/- SEM. The difference in ERK1/2 activation between oxLDL-treated and vehicle-treated cells was determined by Student's t-test.

We confirmed that the antibodies for phosphorylated and total ERK1/2 visualize only the indicated two bands equivalent to the molecular weights of 42 and 44kDa.

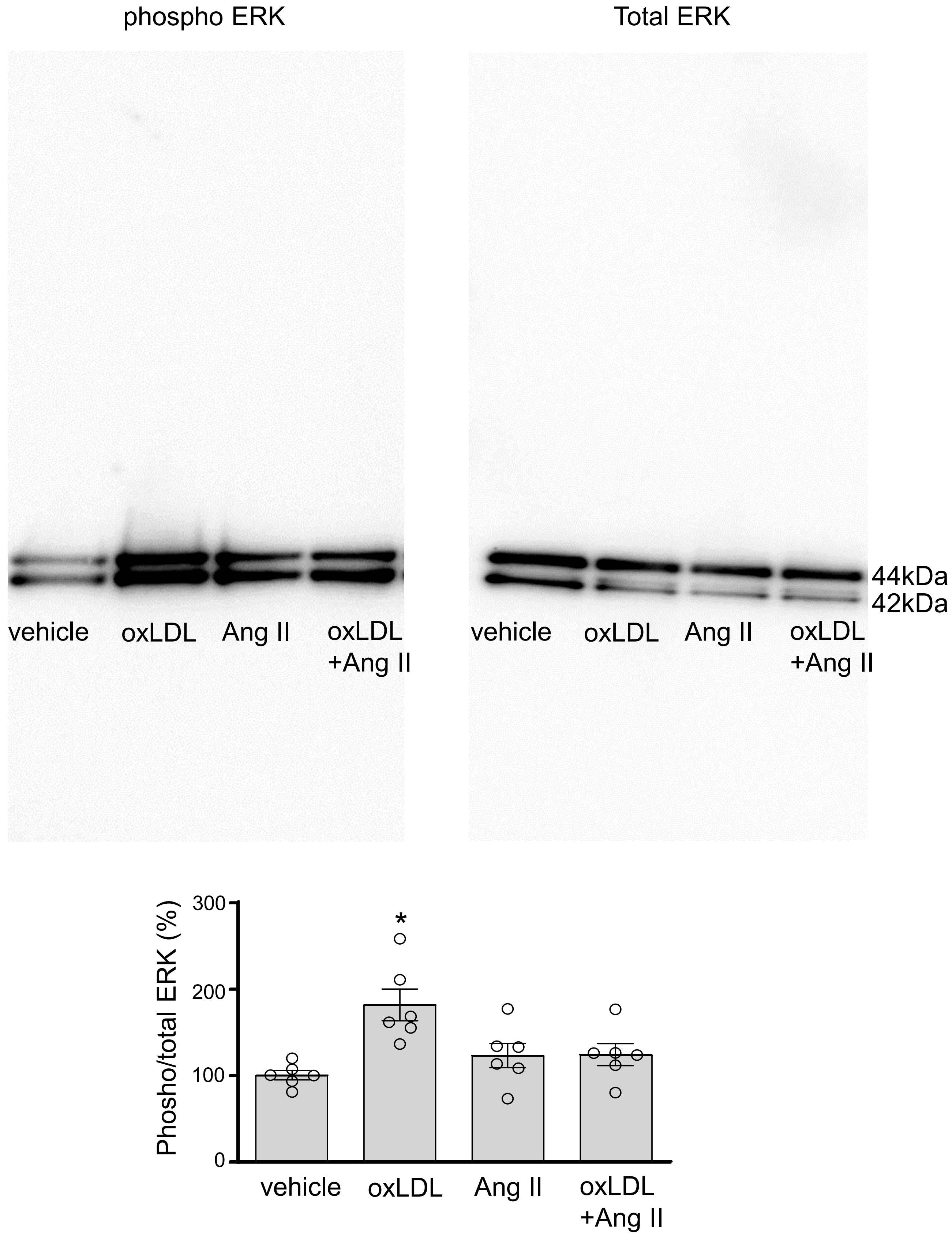

Figure S9: The effect of Ang II and oxLDL on cellular signaling was not additive but rather competitive in HUVECs. Related to Figure 6.

Immunoblotting for detecting phosphorylation of extracellular signal-regulated kinase 1/2 in human umbilical vein endothelial cells (HUVECs). Cells were stimulated with vehicle, 20μg/ml oxLDL, 10^{-7}M Ang II or oxLDL in combination with Ang II for 10 min. (Upper panel) Representative immunoblots of phosphorylated ERK1/2 and total ERK1/2. (Lower panel) Quantification of ERK 1/2 activation by densitometric analysis of phosphorylated/total ERK 1/2. The average activation after vehicle treatment following vehicle pretreatment was set at 100%. Data are represented as mean +/- SEM. Differences were determined by one-way ANOVA with Bonferroni correction.

*$p < 0.01$ vs. control

We confirmed that the antibodies for phosphorylated and total ERK1/2 visualize only the indicated two bands equivalent to the molecular weights of 42 and 44kDa.

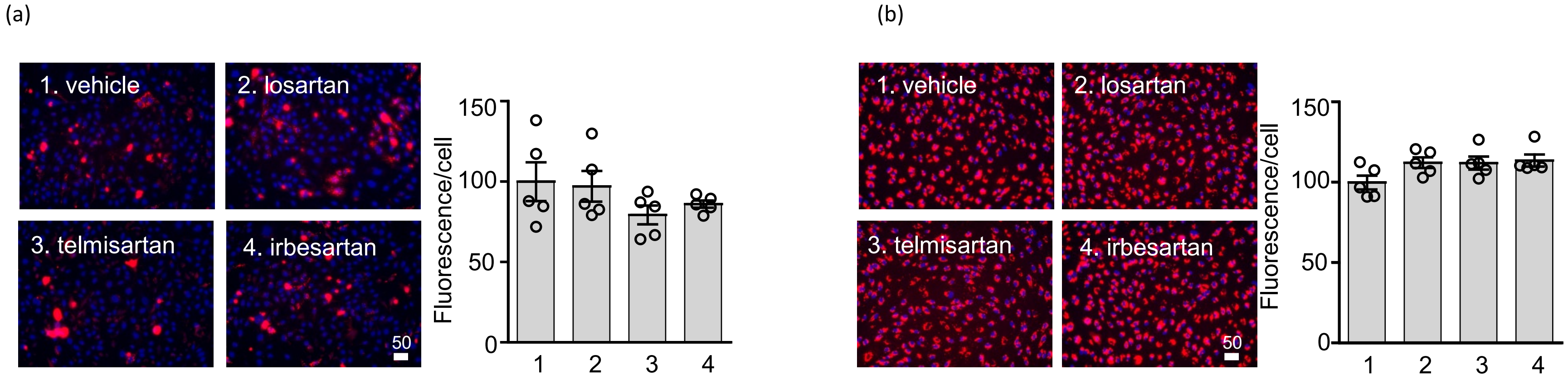

Figure S10: ARBs had no effect on oxLDL uptake in CHO or HUVECs. Related to Figure 6.

Intracellular uptake of 2 μg/ml Dil-labeled oxLDL in (a) CHO-LOX-1-AT1 or (b) HUVECs pretreated with vehicle or 10 μM AT1 blockers (ARBs) (n = 5, each). The graph indicates the fluorescence/number of nuclei, and the average value of the group at the far left was normalized to 100%. Data are represented as mean +/- SEM. The differences were determined by one-way ANOVA.

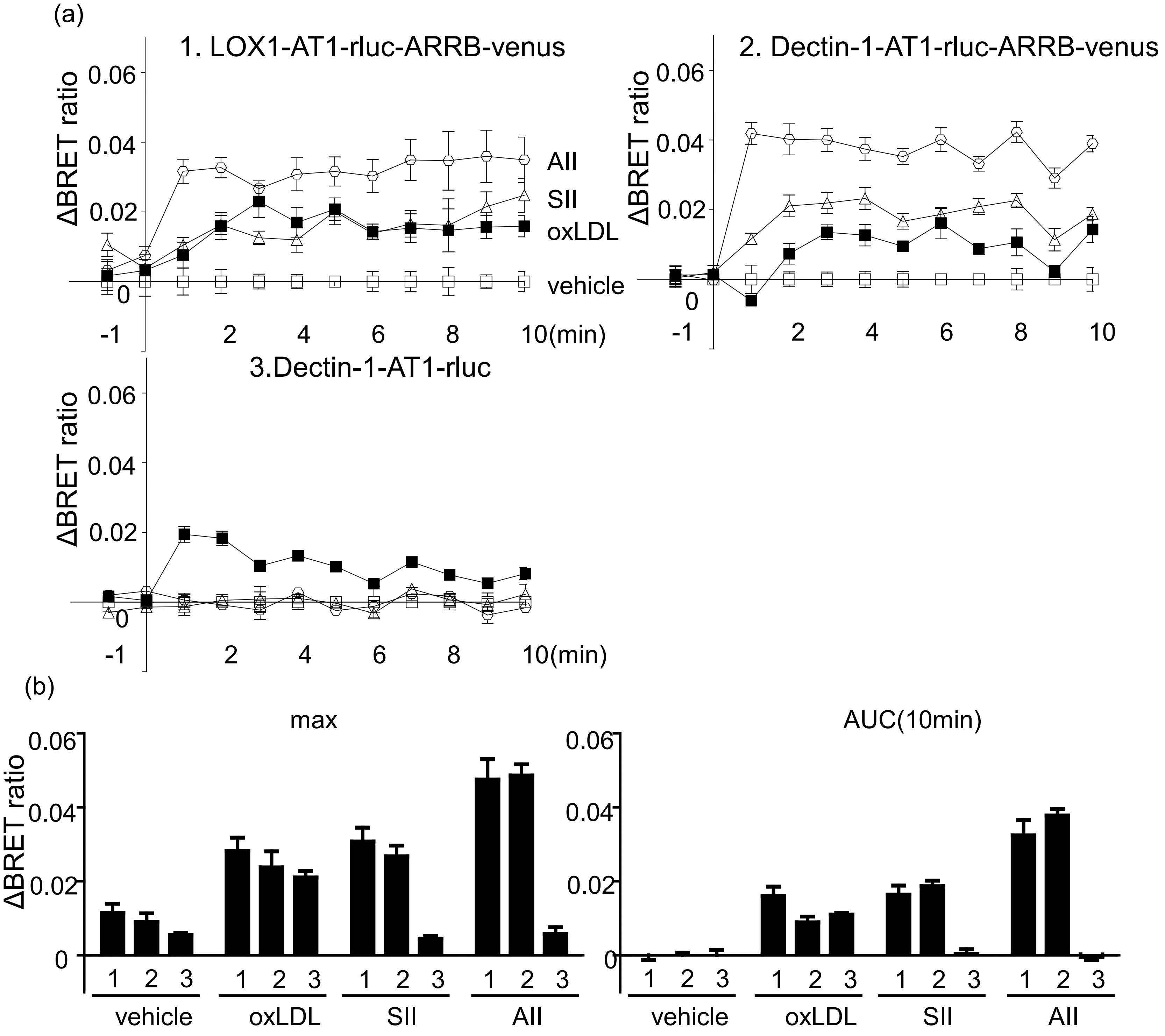

Figure S11:oxLDL-induced BRET between AT1-Rluc8 and β-arrestin 2 (ARRB2)-mVenus in CHO-K1 cells in the presence of LOX-1. Related to Figure 4.

(a) Kinetic data for real-time BRET assay. Coelenterazine h was added before measurement, and each reagent (vehicle, angiotensin II (Ang II) (10-5 M), SII, an arrestin-biased agonist of AT1 (10-5 M), or oxLDL (100 μg/ml)) was added immediately after 0 min. The ΔBRET ratio adjusted by that in vehicle treatment was calculated as described in the Online Methods (n = 8 in 1. LOX1-AT1-rluc-ARRB2-venus, and 2. Dectin-1-AT1-rluc-ARRB2-venus, n = 4 in 3. Dectin-1-AT1-rluc (without ARRB2-venus)).

(b) Comparison of ΔBRET ratios among cells transfected with 1. LOX1-AT1-rluc-ARRB2-venus, 2. Dectin-1-AT1-rluc-ARRB2-venus, and 3. Dectin-1-AT1-rluc. Max BRET ratio and area under the curve (AUC) at 1–10 min are presented. There were no significant differences in max and AUC of the ΔBRET ratio among the transfection in response to oxLDL. Data are represented as mean +/- SEM. The differences were determined by one-way ANOVA.
